# Targeting MET Signalling Activated by CPNE3‐RACK1 Interaction Through VWFA Domain to Suppress Lung Cancer Progression

**DOI:** 10.1111/jcmm.70926

**Published:** 2025-11-05

**Authors:** Xin Cai, Jian Zhao, Chenkang Ma, Min Jiao, Weijie Zhang, Anqi Wang, Jianjun Li, Jianjie Zhu, Yuanyuan Zeng, Chuanyong Mu, Jian‐An Huang, Zeyi Liu

**Affiliations:** ^1^ Department of Pulmonary and Critical Care Medicine The First Affiliated Hospital of Soochow University Suzhou China; ^2^ Institute of Respiratory Diseases Soochow University Suzhou China; ^3^ Suzhou Key Laboratory for Respiratory Diseases Suzhou China

**Keywords:** CPNE3, JNJ‐38877605, MET, NSCLC, RACK1

## Abstract

Despite advances in diagnosis and treatment, the prognosis of non‐small cell lung cancer (NSCLC) remains poor. Therefore, it is urgent to identify potential molecular targets. In this study, we investigated the function and internal mechanism of CPNE3 in the malignant biological behaviour via RACK1/c‐MET signalling in NSCLC, and explored the feasibility of the MET inhibitor in NSCLC treatment. The expression of CPNE3 in normal tissues and lung cancer tissues was compared using a public database. The function of CPNE3 was investigated using CCK‐8 assays, clonogenic assays, EdU assays, Transwell assays and cell cycle analysis. Western blotting was used to detect the protein expression. The interaction between CPNE3 and RACK1 was examined by immunofluorescence staining and co‐immunoprecipitation (co‐IP). The in vitro and in vivo functions of the MET inhibitor JNJ‐38877605 were investigated. CPNE3 is overexpressed in NSCLC and facilitates tumorigenesis and metastasis by interacting with RACK1 through the VWFA domain, which further induces the activation of MET signalling. Accordingly, this process could be suppressed by the MET inhibitor and RACK1 knockdown in vitro and in vivo. CPNE3 is highly expressed in NSCLC and can promote the proliferation and migration of tumour cells. CPNE3 could interact with RACK1 through the VWFA domain and activate MET signalling. These findings may provide new insights into the development of novel therapeutic strategies for NSCLC.

**Abbreviations**
co‐IP: Co‐immunoprecipitation
CPNE3: Calcium‐dependent phospholipid‐binding protein‐III
EMT: Epithelial–mesenchymal transition
FAK: Focal adhesion kinase
HGF: Hepatocyte growth factor
NSCLC: Non‐small cell lung cancer
RACK1: Receptor for activated C kinase l

## 1 Introduction

Lung cancer is the leading cause of cancer‐related death worldwide [1, 2]. In recent years, due to the popularisation of early screening and the development of multiple therapy strategies, the treatment of non‐small cell lung cancer (NSCLC) has greatly improved [3, 4]. In addition, scientists have discovered that nanomaterials can enhance the targeting ability and therapeutic efficacy of anti‐tumour drugs. They can even serve as adjuvants in radiotherapy, optimising treatment outcomes and minimising side effects [5, 6]. However, because of recurrence, metastasis and drug resistance, the overall survival and prognosis of patients with NSCLC remain poor [3]. Therefore, further exploration of key biomarkers affecting the occurrence and development of NSCLC is of great significance for finding new therapeutic targets and improving therapeutic efficacy.

Copine (CPNE) proteins, members of a family of calcium‐dependent phospholipid‐binding proteins, are highly conserved from plants to humans [7, 8]. The CPNE family contains nine members (CPNE1 to CPNE9), all of which have 2 tandem C2 domains (C2 1 and C2 2) at the N‐terminus and an A domain at the C‐terminus [8, 9, 10]. The A domain, named for von Willebrand factor (VWFA), plasma and extracellular matrix protein, has been studied in integrins and several extracellular matrix proteins and appears to function as a protein‐binding domain [7].

As an important member of this family, CPNE3 also plays an important role in the occurrence and progression of cancer [11, 12]. It has been reported that CPNE3 can induce epithelial–mesenchymal transition (EMT) in breast cancer by activating ErbB2 protein and then can promote the invasion and migration of breast cancer cells [13]. In colorectal cancer, relevant studies have found that patients with low CPNE3 levels in exosomes have significantly better disease‐free survival and overall survival, suggesting that CPNE3 may be used as a biomarker to diagnose and evaluate the prognosis of colorectal cancer [14, 15]. In hepatocellular carcinoma, studies have confirmed that knockdown of CPNE3 expression can enhance the sensitivity of hepatocellular carcinoma cells to the molecularly targeted drug sorafenib [16, 17]. In glioblastoma, studies have shown that CPNE3 can promote the proliferation, migration and invasion of tumour cells by activating the PI3K/AKT pathway [18, 19]. CPNE3 can interact with RACK1 and activate the focal adhesion kinase (FAK) signalling pathway to promote the proliferation and metastasis of NSCLC [20]. The role of CPNE3 in various cancers has been confirmed, but the specific molecular mechanism of CPNE3 in NSCLC remains to be further investigated. Therefore, this study aimed to explore the role of CPNE3 in NSCLC and its specific mechanism through multiple experimental assays in vitro and in vivo.

Our previous studies have shown that CPNE3 can activate the epidermal growth factor receptor in A549 cells. However, the internal mechanism in this process remains unknown. Therefore, the purpose of this study was to determine the role of CPNE3 in the proliferation and metastasis of NSCLC and to clarify the internal mechanism of CPNE3 in biological processes in NSCLC.

Receptor for activated C kinase l (RACK1) belongs to the tryptophan‐aspartate repeat (WD‐repeat) family and is a protein homologue of a heterotrimeric G‐protein β subunit [21, 22]. Studies have shown that the expression of RACK1 is related to EMT and can promote the invasion and metastasis of tumour cells [23, 24]. In addition, RACK1 can regulate its function through interactions with several key proteins, thus affecting the occurrence and development of various tumours [24]. Based on previous research and experimental results, we hypothesised that RACK1 may be an important molecule that mediates CPNE3 function and promotes malignant biological behaviours in NSCLC.

The *MET* gene is located on the long arm of human Chromosome 7 (7q21–31), and its protein product is c‐MET receptor tyrosine kinase [25, 26, 27]. The only ligand of c‐MET found thus far is hepatocyte growth factor (HGF) [27, 28]. Under physiological conditions, normal expression of c‐MET pathway components can promote tissue differentiation and repair [25]. In contrast, abnormal activation of the c‐MET pathway in tumour tissues can promote the proliferation and metastasis of tumour cells [29]. Based on the diversity and importance of c‐MET gene variants, drugs targeting c‐MET have good application prospects [30]. Currently, *MET* gene variants known as therapeutic targets for NSCLC mainly include MET 14 exon jump mutations and *MET* amplification. Crizotinib, cabozantinib, sevitinib and other drugs have been marketed or put into clinical trials [31]. In this study, we used a small molecule inhibitor of c‐MET, JNJ‐38877605, which has been used in many studies [32]. JNJ‐38877605 is a small molecule ATP‐competitive c‐MET inhibitor with an IC50 of 4 nM [33]. Furthermore, JNJ‐38877605 acts more than 600 times more selectively on c‐MET than on more than 200 other tyrosine and serine kinases, effectively inhibiting HGF‐stimulated and constitutionally activated c‐MET phosphorylation [34].

In this study, RTK protein chip detection results showed that abnormally high expression of CPNE3 could also lead to an increase in c‐MET phosphorylation levels, so we speculated that c‐MET signalling is the key signalling pathway activated by the CPNE3/RACK1 complex. Therefore, this study will explore the mechanism of CPNE3/RACK1 complex activation of the c‐MET signalling pathway to identify new strategies targeting the c‐MET signalling pathway in the clinical treatment of NSCLC.

## 2 Materials and Methods

### 2.1 Cell Lines and Cell Culture

The NSCLC cell lines A549, H1299, H460, PC‐9, HCC827, H1975, H1650, H1703, H226, H520 and HEK‐293T were purchased from the Cell Bank of the Chinese Academy of Sciences (Shanghai, China). Cells were incubated at 37°C in a humidified air atmosphere containing 5% carbon dioxide in RPMI 1640 medium with 10% foetal bovine serum (FBS), 100 μg/mL penicillin (Sigma–Aldrich, USA) and 100 μg/mL streptomycin (Sigma–Aldrich, USA). All cell lines were mycoplasma‐free and authenticated by quality examinations of their morphology and growth profiles.

### 2.2 Clinical NSCLC Tissue Samples

Thirty paired patient samples of NSCLC tissues and matched adjacent non‐cancerous tissues were obtained from the First Affiliated Hospital of Soochow University between 2017 and 2020. The patients had been diagnosed with NSCLC based on their histological and pathological characteristics according to the Revised International System for Staging Lung Cancer. Tissue samples were acquired during therapeutic surgery of patients who had not previously received any antitumour therapy. Upon resection, human surgical specimens were immediately frozen in liquid nitrogen and stored at −80°C. Informed consent was obtained from all patients, and this study was approved by the Academic Advisory Board of Soochow University.

### 2.3 RNA Interference

Predesigned short interfering RNA (siRNA) sequences that target different coding regions of CPNE3 or RACK1 were directly synthesised by GenePharma (Suzhou, China). The specific primers for target genes are listed in Table S1. Scrambled siRNA was used as a negative control. Cells were transiently transfected with 100 pmol of siRNA sequences using Lipofectamine 2000 (Invitrogen, USA). After 72 h of transfection, the cells were harvested for further experiments.

### 2.4 RNA Isolation and Quantitative Real‐Time PCR Assays

RNA isolation, cDNA synthesis and real‐time quantitative reverse transcription PCR (qRT–PCR) were performed as previously described [33]. The target sequences for CPNE3 were as follows: forward sequence, GTTTTGGCGCTCAGATACCTCC; reverse sequence, GACAAGACCGATACGCCTCTAC. Gene expression levels were quantified according to the comparative ΔΔCt method, and β‐actin was used as the internal control.

### 2.5 CPNE3 Overexpression Plasmid Construction and Transfection

The CPNE3‐overexpressing plasmid was constructed. The empty vector served as a negative control. HEK293T cells were cultured in Dulbecco's modified Eagle's medium (DMEM) containing 10% FBS at 37°C in a humidified 5% CO_2_ incubator for 48 h. After incubation, the packaged lentiviruses were collected and used to infect A549 and H460 cells. After 2 days, stably CPNE3‐overexpressing cells were selected with 400 μg/mL G418 (Amresco, Solon, OH, USA).

### 2.6 Cell Proliferation Assay

Cells were seeded in 96‐well plates at a density of 2 × 10^3^ cells per well and further grown under normal culture conditions for 24, 48 and 72 h. Cell viability was determined using Cell Counting Kit‐8 (Boster, Wuhan, China) according to the manufacturer's instructions. For the clonogenic assay, cells were diluted in complete culture medium, and 300 cells were reseeded in a 60‐mm plate. After incubation for 7–14 days, depending on the cell growth rate, foci formed by at least 50 cells were stained with 0.1% crystal violet and counted. The experiment was performed in triplicate.

### 2.7 Cell Migration and Invasion Assays

Cell migration and invasion assays were performed in a 24‐well plate with 8‐μm pore size chamber inserts (Corning, USA). A total of 5 × 10^4^ cells (migration assays) and 1 × 10^5^ cells (invasion assays) were inoculated into the upper chambers. For the invasion assays, the wells contained membranes coated with Matrigel (Corning, USA), which was diluted with serum‐free culture medium. In both assays, cells were suspended in 200 μL of RPMI 1640 medium without FBS. In the lower chamber, 800 μL of RPMI 1640 medium supplemented with 10% FBS was added. After 24 h, the cells that remained in the upper chamber were removed with a cotton swab, and the cells that moved to the bottom surface of the membrane were fixed with 100% methanol and stained with 0.1% crystal violet for 20 min. Then, the cells were imaged and counted under a microscope. Assays were conducted independently three times.

### 2.8 Cell Cycle Analysis

According to the instructions of the Cell Cycle Analysis Kit (Beyotime, Shanghai, China), cells were cultured in 6‐well plates and transfected with si‐NC or si‐CPNE3 for 72 h. The cells were then collected, washed with cold phosphate‐buffered saline (PBS), fixed in 70% ethanol at 4°C for 24 h, washed with cold PBS again and stained in a propidium iodide (PI)/RNase A mixture. Next, the cells were kept in the dark at 37°C for 30 min and analysed using a FACSCaliber system (Beckman Coulter, Brea, CA, USA).

### 2.9 Western Blot Analysis

Western blot analysis was performed as previously described [33]. The primary antibodies used in this study were anti‐CPNE3 (11186‐1‐AP) (Protein‐tech, China), anti‐RACK1 (sc‐17,754) (Santa Cruz, USA), anti‐p‐AKT (Ser473) (D9E) (no.: 4060), anti‐AKT (no.: 4685), anti‐p‐ERK (Tr202/Tyr202) (D13.14.4E) (no.: 4370), anti‐ERK (no: 4695) and anti‐β‐actin (no.: 3700) (Cell Signaling Technology, USA). The bands were developed by an electrochemiluminescence reagent, imaged with a Chemi‐Doc XRS+ (Bio‐Rad, USA), and finally quantified with ImageJ software (National Institutes of Health, Bethesda, MD, USA).

### 2.10 Co‐Immunoprecipitation

Co‐immunoprecipitation was performed with A549 and H1299 cells. Equal amounts of protein (3000 μg) were pre‐processed by protein A/G magnetic beads (Thermo Scientific, USA). After 2 h, the lysates were incubated with antibodies at 4°C overnight, followed by overnight incubation with beads. The next day, the beads were gently washed five times with PBS containing 1% Triton X‐100, and the beads were then incubated with 2× protein loading buffer at 100°C for 10 min. IgG‐bound, CPNE3‐bound or RACK1‐bound proteins were separated using SDS–PAGE and subjected to western blot analysis.

### 2.11 Immunofluorescence Staining

Cells were seeded in 12‐well plates containing preinserted glass slides. Then, the cells were washed with PBS 24 h later at a confluence of 40%–50%. Cells were fixed with 4% paraformaldehyde for 30 min afterwards, followed by permeabilisation with 0.5% Triton X‐100 solution for an additional 20 min. Next, 5% bovine serum albumin was added to function as the blocking buffer. The primary antibodies, anti‐CPNE3 and anti‐RACK1, and corresponding secondary antibodies conjugated to Cy3 and FITC were used successively.

### 2.12 Animal Experiments

Female BALB/C nude mice aged 4–6 weeks and weighing 16–20 g were selected as experimental animals. The animals were provided by Shanghai Slack Laboratory Animal Co. Lentivirus‐infected A549 cells were used to construct a cell line with stable CPNE3 overexpression. The control cell lines and CPNE3‐overexpressing cell lines were cultured, digested when the cells were in the logarithmic growth phase, and then centrifuged and resuspended using serum‐free medium. The cell concentration was determined and adjusted to 4 × 10^6^/50 μL; the same volume of matrix glue mix was added, and the samples were quickly placed on ice for use. The cells were resuspended under aseptic conditions, and then, 100 μL of cell suspension was removed with a 1‐mL insulin syringe and inoculated into both sides of the nude mice. Tumour formation in the mice was observed after the inoculation 5–7 days later. The time it took for tumours to form was closely recorded, and then, the values of tumour long diameter and short diameter were measured with Vernier callipers every 2 days. The volume was calculated according to the following formula: V(mm^3^) = a × b^2^/2, where a is the long diameter and b is the short diameter. When the tumour volume reached 100–150 mm^3^, DMSO or JNJ‐38877605 was given by gavage at 50 mg/kg/d. At the same time, the weight and growth state of nude mice were observed. When the tumour volume reached 1000 mm^3^, the nude mice were sacrificed, the subcutaneous tumour bodies of the nude mice were photographed and weighed, the tissue protein was ground, and the expression level of related proteins was detected by western blotting. All animal experiments were carried out in accordance with the Guide for the Care and Use of Experimental Animals Center of Soochow University.

### 2.13 Interactive Docking Prediction of Protein–Protein Complexes

The protein interaction between CPNE3 and RACK1 was investigated using the molecular docking dynamic ZDOCK website (https://zdock.umassmed.edu/) [35].

### 2.14 Statistical Analysis

All the data are expressed as the mean ± standard deviation (mean ± SD). SPSS 19.0 software and GraphPad 8.0 were used for statistical analysis of the experimental data; Student's *t* test was used for pairwise comparisons, and One‐way ANOVA was performed for experiments with ≥ 3 independent groups. All experiments were performed with three biological replicates (*n* = 3) from independently prepared cell cultures, whereas technical replicates were averaged prior to analysis to ensure data consistency.

## 3 Results

### 3.1 CPNE3 Is Overexpressed in Lung Cancer and Correlates With Poor Survival

**FIGURE 1:**
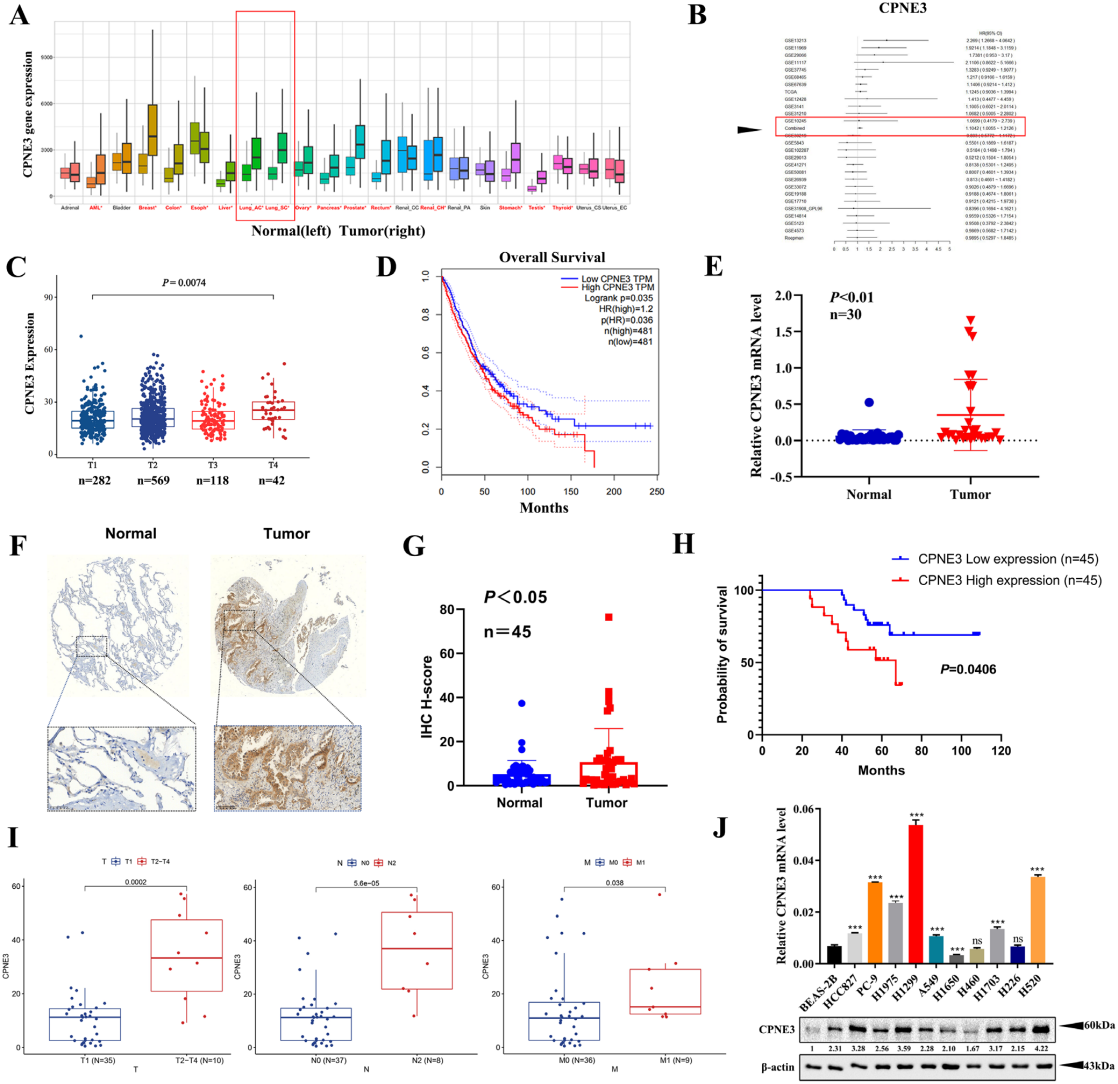
CPNE3 expression is elevated in lung cancer and correlates with poor survival. (A) Comparison of the expression of CPNE3 between cancer tissue and normal tissue in the TCGA database. (B) Analysis of the role of CPNE3 in NSCLC through the OSluca database. (C) Download of CPNE3 expression level and patient clinical information from the TCGA database and comparison of the relationship between CPNE3 expression level and patient T stage. (D) Analysis of the relationship between the expression level of CPNE3 and the overall survival rate of patients in the GEPIA database. (E) Comparison of the mRNA levels of CPNE3 in lung cancer tissue and adjacent tissues using qRT–PCR. (F) Immunohistochemistry (IHC) to detect CPNE3 expression in lung adenocarcinoma tissues and adjacent non‐cancerous tissues in 45 pairs LUAD samples. (G) IHC quantification. (H) Survival curve of 45 patients. (I) CPNE3 expression levels in early‐stage (T1, *n* = 35) versus advanced‐stage (T2–T4, *n* = 10) tumours. The T2–T4 group exhibited significantly higher CPNE3 expression (*p* = 0.0002). Comparison of CPNE3 expression between patients without lymph node metastasis (N0, *n* = 37) and those with advanced nodal involvement (N2, *n* = 8). CPNE3 levels were significantly elevated in the N2 group (*p* = 5.6 × 10^−5^). CPNE3 expression in non‐metastatic (M0, *n* = 36) versus metastatic (M1, *n* = 9) samples. The M1 group showed significantly higher expression (*p* = 0.038). (J) mRNA and protein expression levels of CPNE3 in 10 different NSCLC cell lines and the human normal bronchial epithelial cell line BEAS‐2B were measured by qRT–PCR and western blotting. ns *p* > 0.05; ***p < 0.001.

We first investigated the possible role of CPNE3 in non‐small cell lung cancer through numerous bioanalysis sites. The Kaplan–Meier plotter database showed that CPNE3 is upregulated in a variety of cancers (https://kmplot.com/analysis/, Figure 1A). The OSluca database showed that CPNE3 plays a tumour‐promoting role in LUAD and LUSC (https://bioinfo.henu.edu.cn/LUCA/LUCAList.jsp, Figure 1B). In addition, analysis of survival data showed that high expression of CPNE3 was correlated with a higher T stage and poor survival in lung cancer patients (http://gepia.cancer‐pku.cn/index.html, Figure 1C,D). Bioinformatic methods based on public datasets also indicated that CPNE3 is highly related to oncogenic biological pathways, including regulation of immune system processes, signal transduction and cell communication (Figure S1). In addition, CPNE3 expression is significantly elevated across tumour stages (I–IV) compared with normal tissues (Kruskal–Wallis test, *p* = 1.15e−10, Figure S2). We collected 30 pairs of fresh cancer tissues and adjacent tissues from patients with NSCLC after surgery, and we extracted RNA from the tissues and performed qRT–PCR to detect the mRNA expression of CPNE3. The results indicated that the expression of CPNE3 in NSCLC tissues was higher than that in adjacent tissues (Figure 1E). Moreover, we examined the expression of CPNE3 in 45 pairs of lung adenocarcinoma (LUAD) tissues and matched adjacent non‐tumour tissues using tissue microarray technology. Immunohistochemical staining and scoring results revealed that CPNE3 was significantly upregulated in LUAD tissues (Figure 1F,G). Survival analysis indicated that patients with high CPNE3 expression had a shorter overall survival (Figure 1H). CPNE3 expression is significantly associated with tumour stage, lymph node metastasis and distant metastasis in LUAD (Figure 1I). For further investigation, we detected the expression of CPNE3 in NSCLC cell lines and found that the expression of CPNE3 in lung cancer cell lines (HCC827, PC‐9, H1299, A549, H1650, H460, H1703, H226 and H520) was higher than that in a normal lung epithelial cell line (BEAS‐2B) using western blotting and qRT–PCR (Figure 1J).

### 3.2 The Role of CPNE3 in Mediating NSCLC Cell Proliferation, Migration and Invasion

**FIGURE 2:**
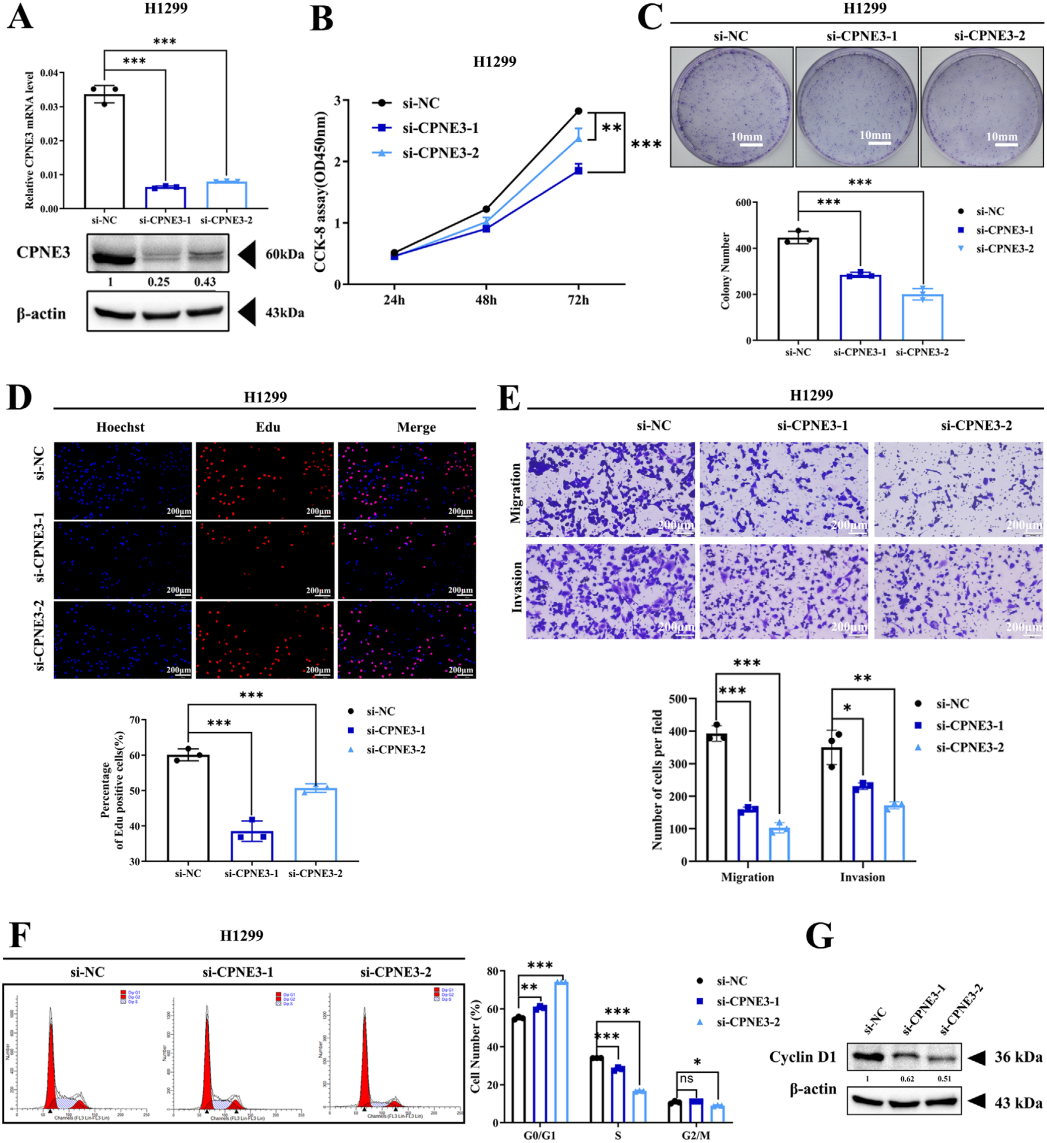
Interfering with CPNE3 expression can inhibit the proliferation, migration and invasion of NSCLC cells and block the cells in the G0/G1 phase. (A) Western blotting and qRT–PCR verification of the effect of interfering with CPNE3. (B) CCK‐8 experiment to detect cell proliferation ability. (C) Clonogenesis assay for detecting cell colony formation ability. (D) EdU experiment to detect cell proliferation ability. (E) Transwell test was used to detect cell migration and invasion ability. (F) Flow cytometry detection of cell cycle. (G) The expression of cyclin D1 is downregulated after down‐regulation of CPNE3. *p < 0.05; **p < 0.01; ***p < 0.001.

**FIGURE 3:**
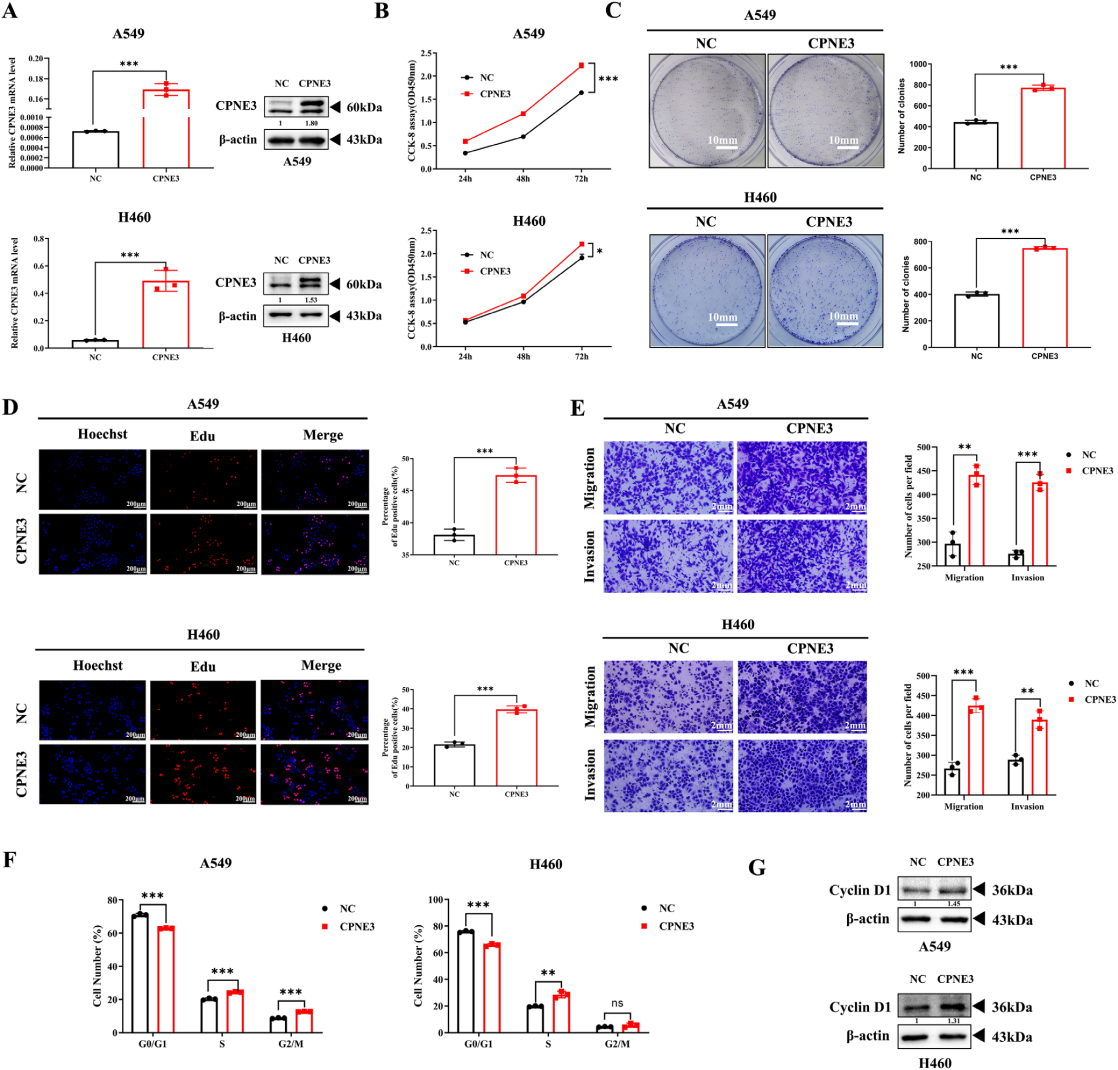
Overexpression of CPNE3 can significantly promote the proliferation, migration and invasion of NSCLC cells and increase the number of S‐phase cells. (A) Western blotting and qRT–PCR validation of the effect of CPNE3 overexpression. (B) CCK‐8 assay to detect cell proliferation. (C) Colony formation assay to detect cell colony formation. (D) EdU assay to detect cell proliferation. (E) Transwell assay to detect cell migration and invasion. (F) Flow cytometry detection of cell cycle. (G) The expression of cyclin D1 is upregulated in CPNE3‐overexpressed A549 and H460 cells. *p < 0.05; **p < 0.01; ***p < 0.001.

The H1299 cell line has a high protein expression level, so we used this cell line for CPNE3 knockdown and subsequent cell function experiments. The expression level of CPNE3 was significantly reduced after transfection with two siRNAs against CPNE3 (Figure 2A). CCK‐8 assays, clonogenic assays and EdU assays were performed to confirm that cell growth was significantly inhibited in CPNE3 knockdown cells compared with control cells after transfection (Figure 2B–D). Transwell assays further demonstrated that the loss of CPNE3 considerably suppressed the migration and invasion of NSCLC cells (Figure 2E). Flow cytometry indicated that after interference with CPNE3, the number of cells in the G0/G1 phase increased, whereas the number of cells in the S (DNA synthesis) phase decreased significantly (Figure 2F, Figure S3). Western blotting showed that the level of Cyclin D1 in CPNE3‐knockdown cells was significantly decreased compared with control cells (Figure 2G). Moreover, we established A549 and H460 cell lines with stable overexpression of CPNE3. These cell lines have moderate baseline CPNE3 expression (Figure 3A). The results showed that cell proliferation, migration and invasion were significantly promoted in cells overexpressing CPNE3 (Figure 3B–E). To minimise cell line‐specific effects and validate the generalisability of our findings, we conducted CPNE3 knockdown experiments in A549 cells (KRAS mutant background) using siRNA and repeated functional assays (CCK‐8, colony formation and Transwell). The results consistently support our earlier findings (Figure S4). In addition, the proportion of cells in the G0/G1 phase was significantly lower, whereas the proportion of cells in the S phase was significantly higher in CPNE3‐overexpressed cells than in control cells (Figure 3F). Western blotting showed that the level of Cyclin D1 in CPNE3‐overexpressed cells was significantly increased compared with control cells (Figure 3G). Collectively, these data strongly suggest that CPNE3 is a crucial oncogene promoting NSCLC progression.

### 3.3 Overexpression of CPNE3 Can Promote Tumour Growth In Vivo

**FIGURE 4:**
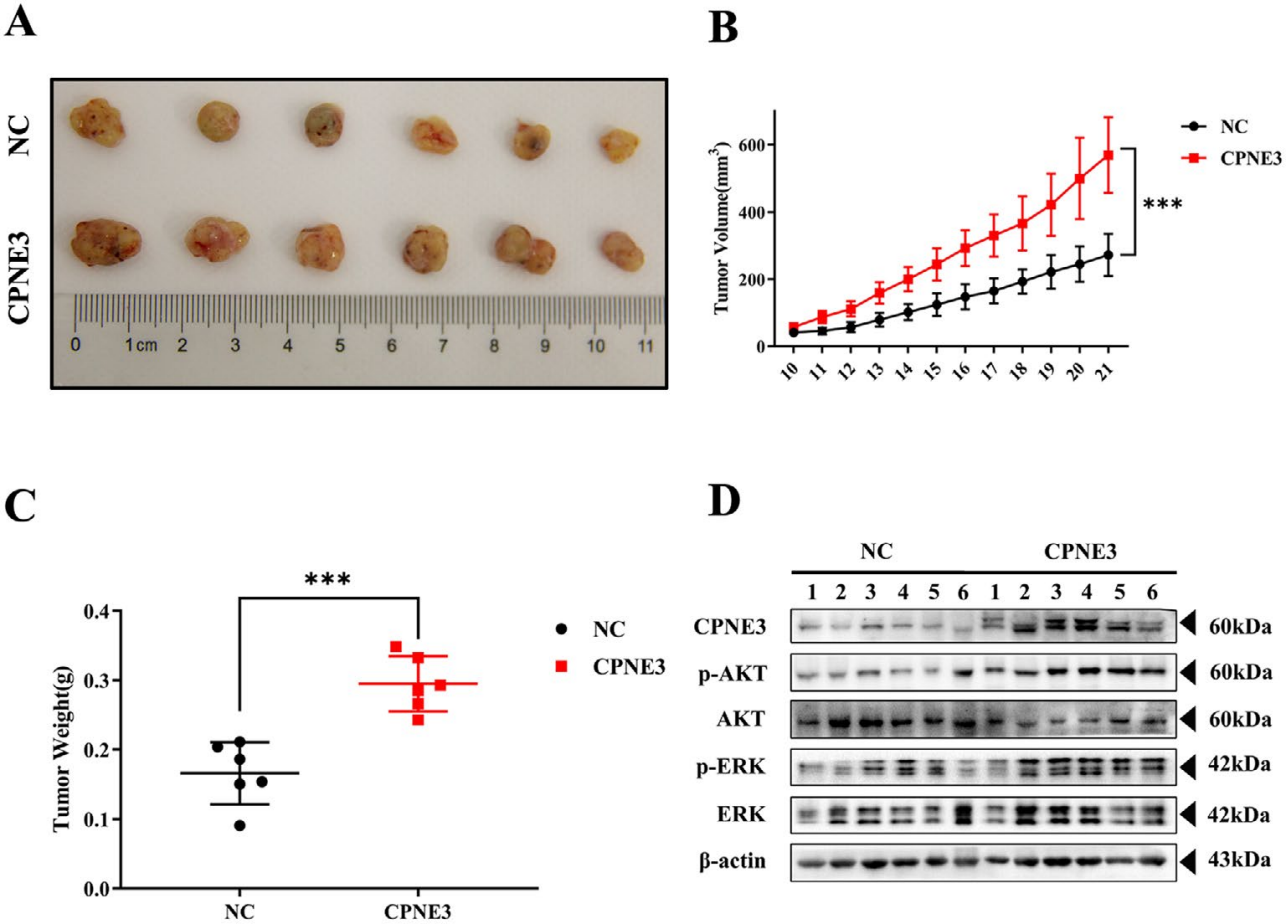
Overexpression of CPNE3 can significantly promote the growth of NSCLC cells in nude mice. (A) Display of subcutaneously transplanted tumour bodies in nude mice. (B) Tumour growth curve. (C) The weight of the tumours. (D) Western blot detection of changes in phosphorylation levels of AKT/ERK in tumour bodies between the overexpression group and the control group. ***p < 0.001.

We further tested the regulatory effect of CPNE3 overexpression on tumour growth in vivo. The A549 cells stably overexpressing CPNE3 were used to inoculate athymic BALB/C nude mice, and the tumours formed with CPNE3‐overexpressed cells were significantly larger in tumour volume than those formed with control cells, and the growth rates were significantly faster (Figure 4A,B). Consistent with these results, tumour weight was significantly higher in CPNE3‐overexpressed tumours than in the controls (Figure 4C). Western blotting showed that the levels of CPNE3, p‐AKT and p‐ERK in CPNE3‐overexpressed tumours were significantly increased compared with control cells (Figure 4D). In conclusion, these data confirmed that CPNE3 expression can affect NSCLC growth in vivo.

### 3.4 CPNE3 Regulates the MET Signalling Pathway in NSCLC Cell Lines

**FIGURE 5:**
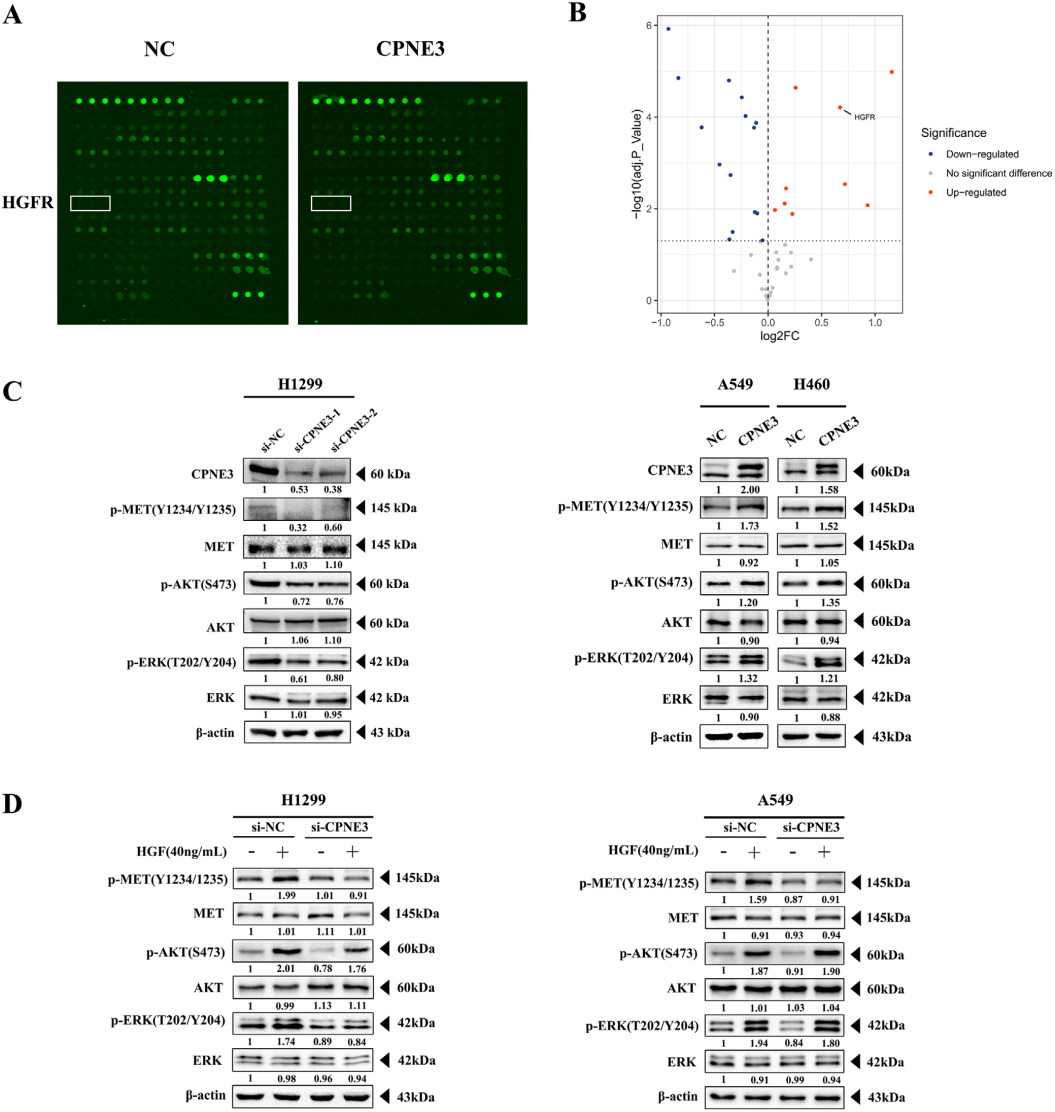
CPNE3 functions by activating the c‐MET signalling pathway. (A, B) RTK protein chip results in CPNE3‐overexpressing cells and control A549 cells. (C) Western blot detection of the changes in c‐MET and downstream AKT/ERK phosphorylation levels between the knockdown CPNE3 group and the control group, as well as the overexpression CPNE3 group and the control group. (D) Western blot detection of the changes in c‐MET and downstream AKT/ERK phosphorylation levels after adding HGF to the knockdown CPNE3 group and control group.

We wanted to determine how CPNE3 plays a significant role in promoting cancer. A human RTK phosphorylation array was performed in vector‐ and CPNE3‐overexpressing cells to identify changes in potential downstream signalling pathways (Figure 5A, Figure S5). The detection results indicated that the phosphorylation level of c‐MET was significantly increased after CPNE3 overexpression, suggesting that CPNE3 plays a role by activating the c‐MET signalling pathway (Figure 5B). Then, western blotting was used to detect the changes in c‐MET phosphorylation levels between the control group, knockdown group and overexpressed group for verification, and the changes in AKT/ERK phosphorylation levels downstream of c‐MET were also detected (Figure 5C). These results showed that the phosphorylation levels of c‐MET and AKT/ERK were significantly increased in the CPNE3 overexpression group. To further verify the mechanism by which CPNE3 acts through the c‐MET signalling pathway, we interfered with CPNE3 in H1299 and A549 cells (using the interference sequence si‐CPNE3‐2) and added exogenous HGF to the CPNE3 interference group and the control group. In this way, the c‐MET signalling pathway was artificially activated, and the c‐MET phosphorylation levels of the CPNE3 group and control group, as well as the phosphorylation levels of AKT/ERK, were determined by western blot detection (Figure 5D). The results suggested that after interference with CPNE3, the activation of c‐MET and downstream AKT/ERK induced by HGF was inhibited, further confirming that CPNE3 can activate the c‐MET signalling pathway.

### 3.5 CPNE3 Interacts With RACK1 in NSCLC Cells

**FIGURE 6:**
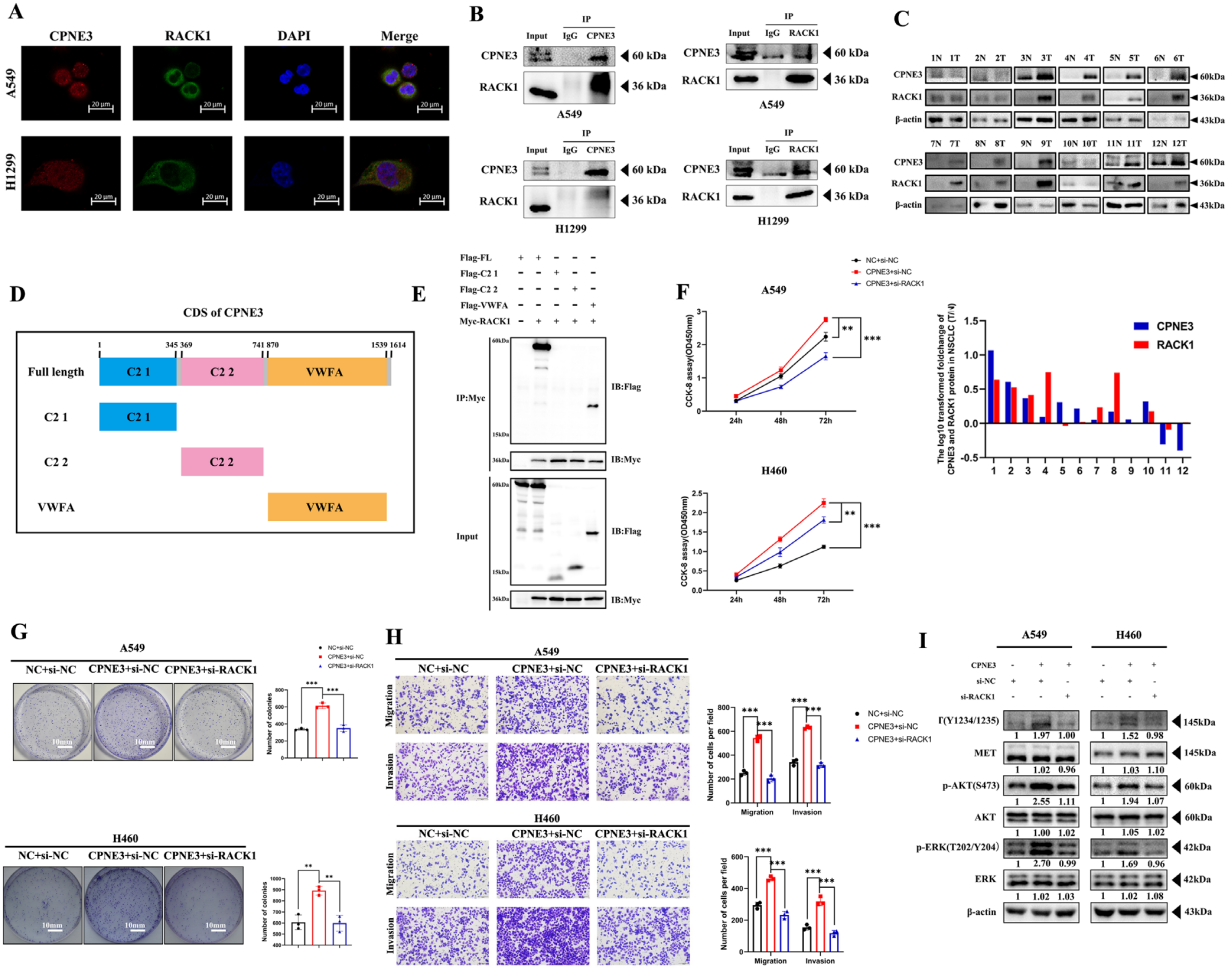
CPNE3 can bind to RACK1 and has a co‐expression relationship with RACK1, thereby promoting the proliferation, migration and invasion of NSCLC cells. (A) Immunofluorescence experiments of CPNE3 and RACK1 in A549 and H1299 cells. (B) Co‐immunoprecipitation test of CPNE3 and RACK1 in A549 and H1299 cells. (C) 12 Detection of the protein expression levels of CPNE3 and RACK1 in NSCLC tissue and corresponding adjacent cancer tissues. The Y‐axis represents the log10 conversion value of the T/N protein expression ratio of CPNE3 and RACK1, whereas the X‐axis represents each sample number. (D) The FL CDS and three truncated CDSs of CPNE3 (denoted FL, C2 1, C2 2 and VWFA) were used for construction of FLAG‐tagged CPNE3 expression vectors. (E) The abovementioned FLAG‐tagged FLAG vectors and MYC‐tagged RACK1 were co‐transfected into HEK293T cells as indicated for 48 h, and then, the cells were subjected to co‐IP analysis to detect the domains of CPNE3 that interact with RACK1. (F) CCK‐8 experiment to detect cell proliferation ability. (G) Colony formation ability of cells detected by colony formation experiment. (H) Transwell assay was used to detect cell migration and invasion ability. (I) Western blot detection of c‐MET phosphorylation level and downstream AKT/ERK phosphorylation level changes. **p < 0.01; ***p < 0.001.

To further determine the underlying mechanism of CPNE3 and MET in NSCLC, we found that the molecular dynamics docking results through the Bioinformatics Analysis website (https://zdock.umassmed.edu/) suggest that CPNE3 can bind to RACK1 (Figure S6). We subsequently conducted a series of experiments to investigate whether the two can bind to each other. Immunofluorescence staining showed that CPNE3 and RACK1 were colocalised in A549 and H1299 cells (Figure 6A). Moreover, we performed co‐immunoprecipitation experiments to confirm the direct relationship between CPNE3 and RACK1 (Figure 6B). To further determine the relationship between CPNE3 and RACK1, we collected 12 pairs of NSCLC lung cancer tissue and corresponding adjacent tissue samples. Western blotting was used to detect the protein expression levels of CPNE3 and RACK1 in these tissues. A total of 10 pairs with high expression of CPNE3 and 11 pairs with high expression of RACK1 were found in cancer tissue. There were 11 pairs of CPNE3 and RACK1 expressed at the same high and low levels, indicating a positive correlation between CPNE3 and RACK1 in NSCLC tissue (Figure 6C). To explore which domain CPNE3 interacts with RACK1, we first generated four various constructs with FLAG tags, which contain a full‐length (FL) coding DNA sequence (CDS) of CPNE3 and three truncated CDSs (i.e., an N‐terminal C2 1 domain, a middle region C2 2 domain and a C‐terminal VWFA domain, Figure 6D). Subsequently, different constructs were co‐transfected into HEK293T cells with MYC‐tagged RACK1 expression vectors. Co‐IP analysis showed that RACK1 coprecipitated with VWFA domains rather than C2 1 and C2 2 of CPNE3 (Figure 6E).

### 3.6 CPNE3 Activates the c‐MET Signalling Pathway by Interacting With RACK1

To confirm that CPNE3 activates the c‐MET signalling pathway by binding to RACK1, we transfected RACK1 siRNAs into A549 and H460 cells stably overexpressing CPNE3. Subsequently, the effects of RACK1 interference on the proliferation, migration and invasion of CPNE3‐overexpressing cells were investigated by CCK‐8, colony formation and Transwell assays (Figure 6F–H). Western blotting was used to detect the changes in c‐MET and AKT/ERK phosphorylation levels of the proteins interfering with the RACK1 group and the control group (Figure 6I). The results showed that interference with RACK1 could significantly inhibit the proliferation, colony formation, migration and invasion ability of CPNE3‐overexpressing cell lines and reduce the phosphorylation levels of c‐MET and AKT/ERK in the CPNE3 overexpression group.

### 3.7 c‐MET Inhibitor JNJ‐38877605 Block CPNE3‐Induced Aberrant Activation

**FIGURE 7:**
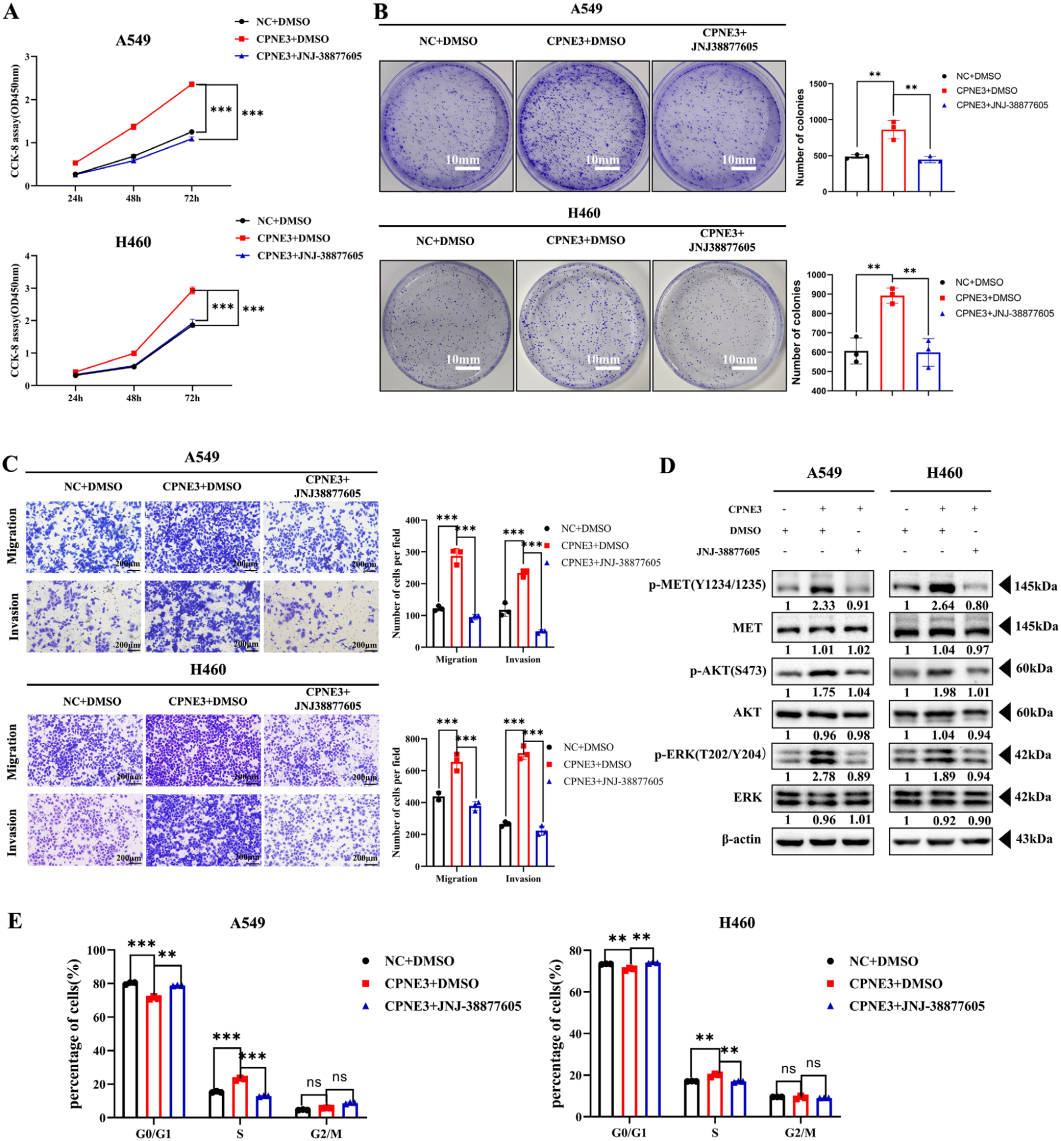
c‐MET inhibitors can inhibit the proliferation, migration and invasion of CPNE3‐overexpressing cells. (A) CCK‐8 experiment to detect cell proliferation ability. (B) Colony formation ability of cells detected by colony formation experiment. (C) Transwell test was used to detect cell migration and invasion ability. (D) Western blot detection of c‐MET phosphorylation level and downstream AKT/ERK phosphorylation level changes. (E) Flow cytometry detection of cell cycle changes. **p < 0.01; ***p < 0.001.

Subsequently, DMSO and JNJ‐38877605 were added to the CPNE3‐overexpressing cell lines A549 and H460 and the control group, respectively. The results showed that JNJ‐38877605 could significantly inhibit the proliferation, colony formation, migration and ability of CPNE3‐overexpressing cell lines (Figure 7A–C). The results of subsequent western blotting also suggested that JNJ‐38877605 could reverse the increase in downstream c‐MET and AKT/ERK phosphorylation levels caused by overexpression of CPNE3 (Figure 7D). Flow cytometry detection of the cell cycle indicated that JNJ‐38877605 could arrest the cells overexpressing CPNE3 in the G0/G1 phase, whereas the proportion of cells in the S phase was reduced (Figure 7E, Figure S7). These results suggest that CPNE3 can promote the proliferation and invasion of NSCLC cells by activating the c‐MET signalling pathway, and this effect can be reversed by c‐MET inhibitors in vitro.

### 3.8 c‐MET Inhibitor Inhibited the Growth of CPNE3‐Overexpressing Cells In Vivo

**FIGURE 8:**
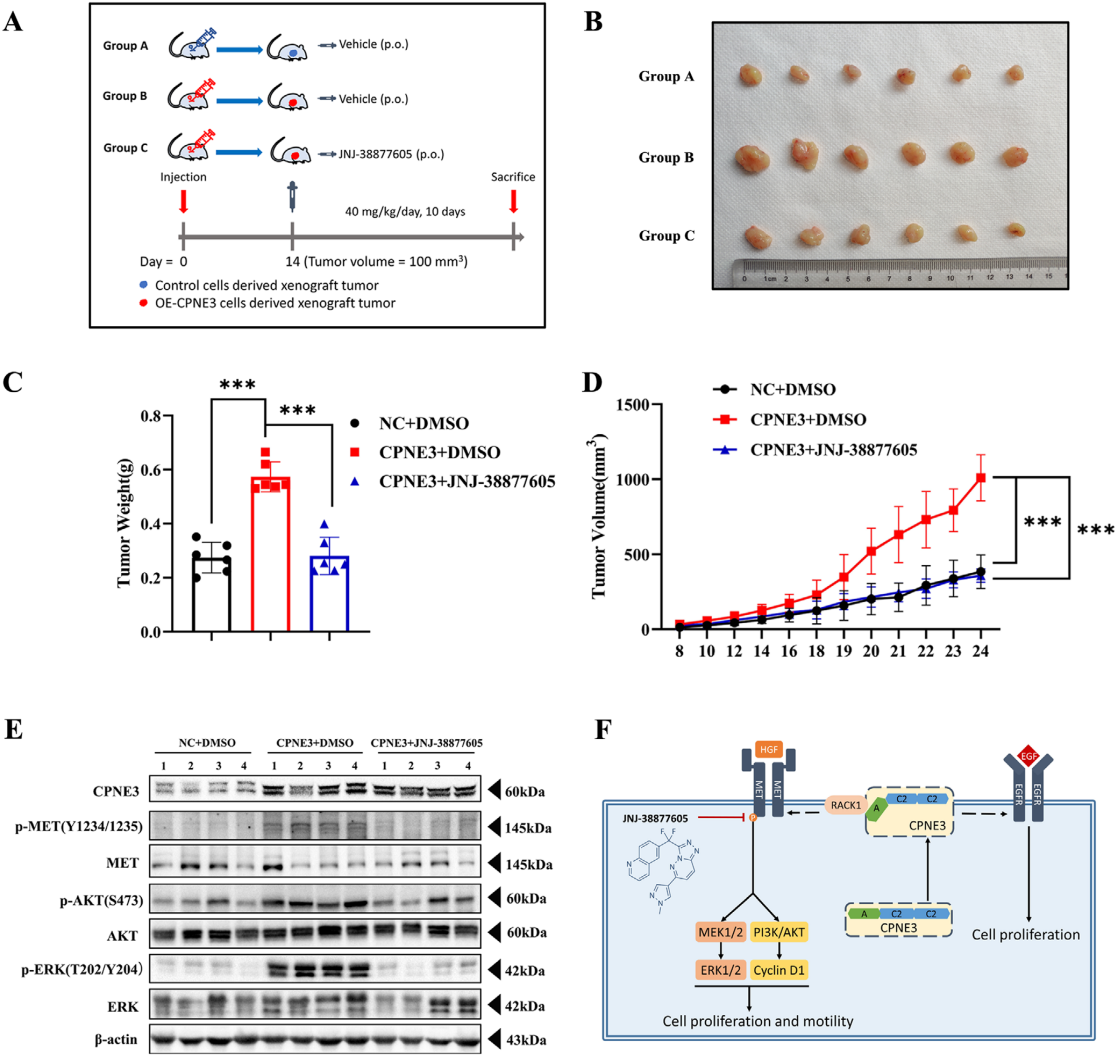
c‐MET inhibitor inhibits the growth of transplanted tumours with CPNE3‐overexpressing cells in nude mice. (A) Schematic diagram of animal experiments. (B) Display of subcutaneously transplanted tumour bodies in nude mice. (C) The weight of the tumour. (D) Tumour growth curve. (E) Western blot detection of the phosphorylation levels of c‐MET and AKT/ERK in tumour cells of the overexpression group and the control group. (F) Schematic illustration of CPNE3 promotes the proliferation and invasion of non‐small cell lung cancer cells via RACK1/c‐MET signalling. ***p < 0.001.

Cell line experiments verified that c‐MET inhibitors can inhibit the proliferation, migration and invasion of CPNE3‐overexpressing cells in vitro. Furthermore, through subcutaneous tumour formation experiments in BALB/c nude mice, mice were divided into three groups: the NC + DMSO group, CPNE3 + DMSO group and CPNE + JNJ‐38877605 group (Figure 8A). After the tumour volume reached 100 mm^3^, the nude mice in the three groups were given gavage treatment, and the weight and tumour volume of the nude mice were monitored daily. The results suggested that the c‐MET inhibitor JNJ‐38877605 could significantly inhibit the growth of transplanted tumours with CPNE3‐overexpressing cells in nude mice (Figure 8B–D). The results of subsequent western blotting experiments indicated that c‐MET and AKT/ERK phosphorylation levels in the tumour were significantly decreased after the c‐MET inhibitor was applied (Figure 8E). In addition, co‐IP assays and western blot assays indicated that CPNE3 could interact with MET and promote oncogenic signalling through a MET‐dependent mechanism (Figure S8). The above results indicated that JNJ‐38877605 could inhibit the growth of CPNE3‐overexpressing cells in vivo.

## 4 Discussion

In this study, we demonstrated that CPNE3 can promote the progression of NSCLC by binding to RACK1 through the VWFA domain and activating the c‐MET signalling pathway, which could be inhibited by using the MET inhibitor JNJ‐38877605.

Previously, it has been reported that CPNE3 may be an important gene involved in the metastasis of non‐small cell lung cancer and has the potential to become a novel therapeutic target [12, 20]. However, the internal mechanism of CPNE3 in mediating tumour progression remains unclear. Several studies have validated the pro‐cancer effects of CPNE3 in various cancers, but there are also studies suggesting that CPNE3 may have antitumour effects [15, 19]. For example, in glioblastoma, a study found that the high expression of CPNE3 can inhibit the invasion, migration and proliferation of glioblastoma cells by inactivating the FAK signalling pathway [18]. The specific role of CPNE3 in glioblastoma is still controversial. Therefore, the specific role of CPNE3 in NSCLC still needs to be further explored. Therefore, we carried out many bioinformatics analyses before starting the experiment. In this study, public data, such as CPNE3 expression levels and patient clinical information, were downloaded from TCGA, GEO and other databases for various analyses. We found that CPNE3 expression is elevated in non‐small cell lung cancer and is predicted to function as an oncogene, which is significantly correlated with TNM staging and poor prognosis in patients. With the support of the data, we believe that CPNE3 plays a cancer‐promoting role in non‐small cell lung cancer and may be a new therapeutic target or an independent prognostic factor for patients.

CPNE3 has been confirmed to play a role as the ligand of ErBb2 [13, 36]. In addition, CPNE3 can affect the FAK signalling pathway [19, 37, 38]. ErBb2 is an important member of the receptor tyrosine kinase family, and FAK is a nonreceptor intracellular tyrosine kinase [36]. We therefore suggest that CPNE3 is closely associated with the family of tyrosine kinases on the cell membrane.

Subsequently, after confirming the carcinogenic effect of CPNE3, we chose to use a protein microarray to detect changes in the phosphorylation level of the tyrosine kinase family. Protein microarray results showed that the phosphorylation level of c‐MET was significantly increased after CPNE3 overexpression, which confirmed that CPNE3 plays a role in activating the c‐MET signalling pathway.

Previous studies have reported that CPNE1, a member of the CPNE3 family, can activate the c‐MET signalling pathway through mutual binding with RACK1, and molecular dynamic docking results suggest that CPNE3 can also bind with RACK1 and activate the c‐MET signalling pathway [39, 40]. It is suggested that CPNE1 and CPNE3 have similarities in that they can both activate the c‐MET signalling pathway, which requires binding with scaffold protein RACK1 to play this role.

We used the UniProt website (https://www.uniprot.org/) and found that CPNE1 and CPNE3 have similar structural domains, both consisting of two C2 domains and one VWFA domain. CPNE1 and other CPNE family members have similar domains, and their carboxyl terminus contains an A domain, named after von Willebrand factor, which is a plasma and extracellular matrix protein structurally related to the extracellular structure of integrins, and its main function is protein binding [15, 20, 41]. The amino terminus contains two C2 domains, which play a crucial role in regulating the binding of calcium and phospholipids [9, 10, 42, 43]. These domains are involved in various cell signalling pathways and cellular processes, including membrane transport, lipid messenger production, GTPase activation and protein phosphorylation. The C2 domain is characterised by an eight‐chain antiparallel β‐sandwich structure and is divided into two different topologies with slightly different positions and connectivity in the β‐chain structure [9, 10]. Our next research plan is to explore whether all CPNE families can be combined with RACK1 through the VWFA domain. CPNE1 and CPNE3 have similar structures and functions and can activate the c‐MET signalling pathway. Both of them bind to RACK1, and whether the CPNE family can bind to RACK1 to exert their functions is worth further investigation.

The important downstream signalling pathway involved in this study is the c‐MET signalling pathway, and the only ligand found for c‐MET is HGF [25, 27]. When the c‐MET pathway is abnormally activated in tumour tissue, it can promote the proliferation and metastasis of tumour cells. Although HGF/c‐MET targeted therapy has facilitated breakthroughs in some cancers, a single therapy targeting HGF/c‐MET has failed to show significant clinical efficacy in most cancers, suggesting that we need to find new therapeutic targets to improve the therapeutic efficiency of inhibitors and provide patients with precise treatment [30]. Our study found that overexpression of CPNE3 significantly increases the phosphorylation level of c‐MET, and the malignant biological behaviour of tumour cells induced by overexpression of CPNE3 can be inhibited by the c‐MET inhibitor JNJ‐38877609, indicating that patients with high expression of CPNE3 are more likely to benefit from treatment with c‐MET inhibitors [30]. In summary, our study demonstrates that high expression of CPNE3 is associated with advanced TNM stage and a poor prognosis in patients with NSCLC, revealing that the interaction between CPNE3 and RACK1 can activate the c‐MET signalling pathway. This study also provides new insights regarding the use of the c‐MET inhibitor JNJ‐38877605 for the precise treatment of patients with NSCLC with high expression of CPNE3.

In conclusion, through the above research, we identified an oncogene, CPNE3. CPNE3 is highly expressed in the tissues of patients with NSCLC, and its high expression is correlated with advanced TNM stage and a poor prognosis for patients. CPNE3 can promote the proliferation, migration and invasion of NSCLC cells. Further studies have shown that CPNE3 can promote the proliferation, migration and invasion of NSCLC cells mainly through binding of the VWFA domain with the scaffold protein RACK1 and activation of the downstream c‐MET signalling pathway, which can be inhibited by the c‐MET inhibitor JNJ‐38877605. In conclusion, this study provides a new theoretical basis for the treatment of NSCLC and the clinical application of c‐MET inhibitors.

## Author Contributions

**Xin Cai:** data curation (equal), writing – original draft (equal). **Jian Zhao:** writing – review and editing (equal). **Chenkang Ma:** writing – review and editing (equal). **Min Jiao:** writing – review and editing (equal). **Weijie Zhang:** writing – review and editing (equal). **Anqi Wang:** writing – review and editing (equal). **Jianjun Li:** writing – review and editing (equal). **Jianjie Zhu:** writing – review and editing (equal). **Yuanyuan Zeng:** writing – review and editing (equal). **Chuanyong Mu:** writing – review and editing (equal). **Jian‐An Huang:** funding acquisition (equal), writing – review and editing (equal). **Zeyi Liu:** writing – review and editing (equal).

## Ethics Statement

The NSCLC tissues were collected with the informed consent of the patients from the First Affiliated Hospital of Soochow University between 2017 and 2020. This study was approved by the Academic Advisory Board of Soochow University.

## Consent

All authors consent to publication.

## Conflicts of Interest

The authors declare no conflicts of interest.

## Supporting information


**Figure S1:** (A) Heatmap of differential expressed mRNAs between CPNE3‐low expression and CPNE3‐high expression group. Data was downloaded from GEO database (https://www.ncbi.nlm.nih.gov/geo/, Microarray ID: GSE31210). (B) Volcano plot for differential expressed mRNAs between CPNE3‐low expression and CPNE3‐high expression group. (C, D) GSEA analysis based on GEO data suggested that the high‐level expression of CPNE3 is related to several oncogenic‐related biological pathways.

**Figure S2:** (A) Box plots depict CPNE3 gene expression levels in normal lung tissue and tumour samples stratified by pathological stage (Stage I–IV). Expression values are shown with each group represented by a distinct colour: green (Normal), yellow (Stage I), blue (Stage II), purple (Stage III), and red (Stage IV). Statistical analysis was performed using the Kruskal–Wallis test (KWp = 1.15 × 10^−10^), indicating significant differences among groups. Post hoc pairwise comparisons were conducted using Dunn's test with adjusted *p* values. Significant differences were observed between Normal and Stage I (*p*_adj = 0.00) and Normal and Stage II (*p*_adj = 0.01).

**Figure S3:** (A) Flow cytometry indicated that after CPNE3‐overexpression, the number of cells in the G0/G1 phase decreased, while the number of cells in the S (DNA synthesis) phase increased significantly.

**Figure S4:** (A) CCK‐8 experiment to detect cell proliferation ability. (B) Clonogenesis assay for detecting cell colony formation ability. (C) Transwell test was used to detect cell migration and invasion ability. *p < 0.05; **p < 0.01; ***p < 0.001.

**Figure S5:** (A) Array map of RayBio Human RTK Phosphorylation Antibody Array G‐series. (B) ELISA assay was performed to detect the effect of CPNE3 expression on HGF concentration in cell supernatant.

**Figure S6:** (A) The protein interaction between CPNE3 and RACKl was investigated using the molecular docking dynamic ZDOCK website (https://zdock.umassmed.edu/). (B) Structure of the vector plasmid used in this study.

**Figure S7:** (A) Flow cytometry indicated that JNJ‐38877605 could arrest the cells overexpressing CPNE3 in the G0/G1 phase, while the proportion of cells in the S phase was reduced.

**Figure S8:** (A) Co‐IP assays demonstrate complex formation between CPNE3 and MET. Lysates from A549 were immunoprecipitated with anti‐CPNE3 or anti‐MET antibodies, followed by immunoblotting with the indicated antibodies. Both forward (IP: CPNE3) and reverse (IP: MET) Co‐IPs confirm specific interaction between CPNE3 and MET, while control IgG shows no precipitation. (B) MET inhibitor JNJ‐38877605 partially reverses CPNE3‐induced AKT and ERK activation. CPNE3‐overexpressing cells were treated with JNJ‐38877605, and phosphorylation levels of AKT and ERK were analysed by Western blot. The results indicate that CPNE3‐driven signalling is dependent on MET activity.

**Table S1:** Sequences of siRNAs.

**Table S2:** Relationship between clinical characteristics and *CPNE3* mRNA expression.

**Table S3:** Relationship between clinical characteristics and CPNE3 protein expression.

## Data Availability

The datasets generated and/or analysed during the current study are available from the corresponding author upon reasonable request.
