# Supplementary figures and images for: Targeting MET Signalling Activated by CPNE3‐RACK1 Interaction Through VWFA Domain to Suppress Lung Cancer Progression
Source: J Cell Mol Med. 2025 Nov 5;29(21):e70926. doi: 10.1111/jcmm.70926 (PMC12587306; doi:10.1111/jcmm.70926)

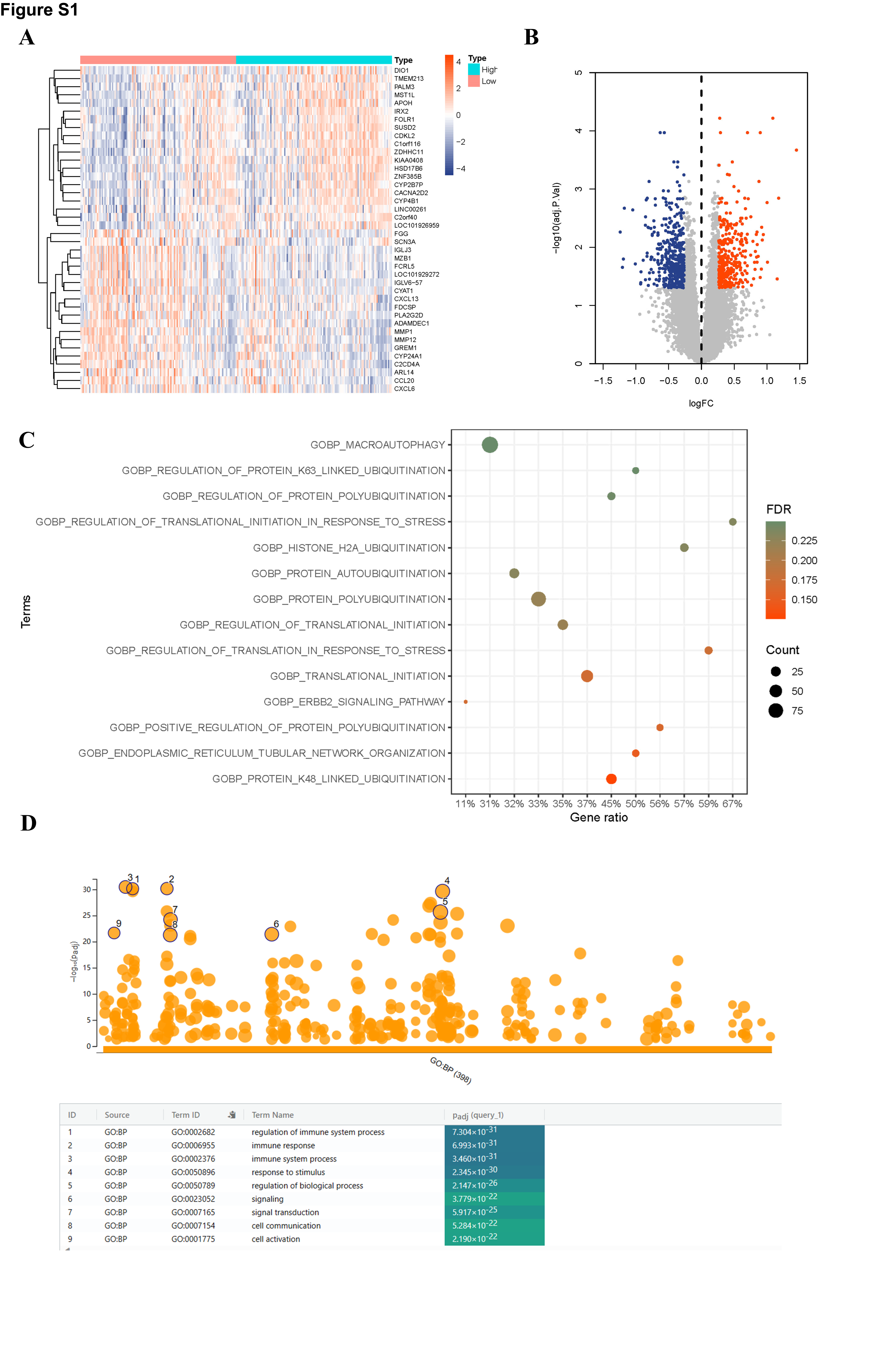

Supplement: Supplementary file 1 — Figure S1: (A) Heatmap of differential expressed mRNAs between CPNE3‐low expression and CPNE3‐high expression group. Data was downloaded from GEO database (https://www.ncbi.nlm.nih.gov/geo/, Microarray ID: GSE31210). (B) Volcano plot for differential expressed mRNAs between CPNE3‐low expression and CPNE3‐high expression group. (C, D) GSEA analysis based on GEO data suggested that the high‐level expression of CPNE3 is related to several oncogenic‐related biological pathways. [file JCMM-29-e70926-s007.tif]

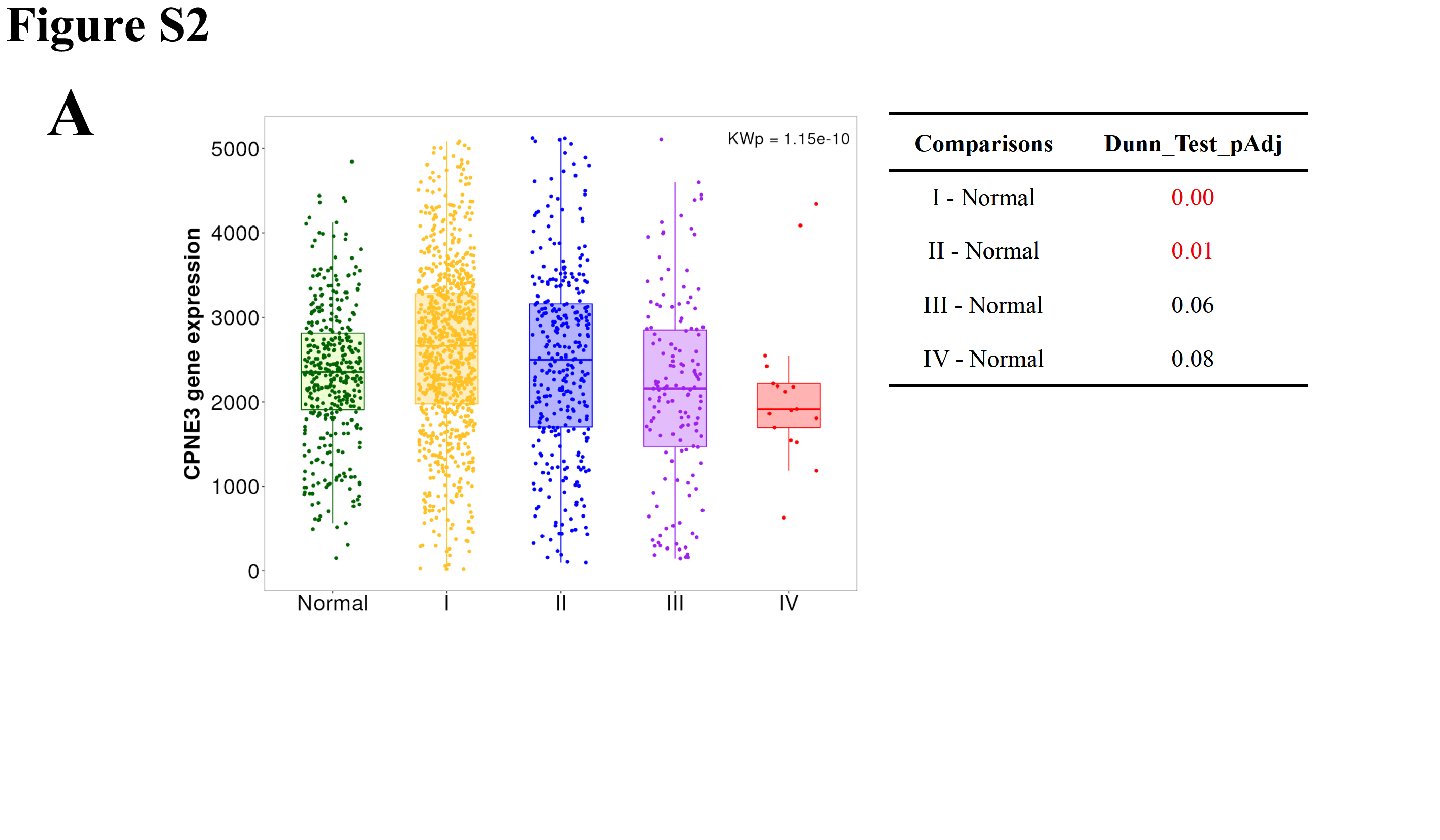

Supplement: Supplementary file 2 — Figure S2: (A) Box plots depict CPNE3 gene expression levels in normal lung tissue and tumour samples stratified by pathological stage (Stage I–IV). Expression values are shown with each group represented by a distinct colour: green (Normal), yellow (Stage I), blue (Stage II), purple (Stage III), and red (Stage IV). Statistical analysis was performed using the Kruskal–Wallis test (KWp = 1.15 × 10^−10^), indicating significant differences among groups. Post hoc pairwise comparisons were conducted using Dunn's test with adjusted p values. Significant differences were observed between Normal and Stage I (p_adj = 0.00) and Normal and Stage II (p_adj = 0.01). [file JCMM-29-e70926-s008.tif]

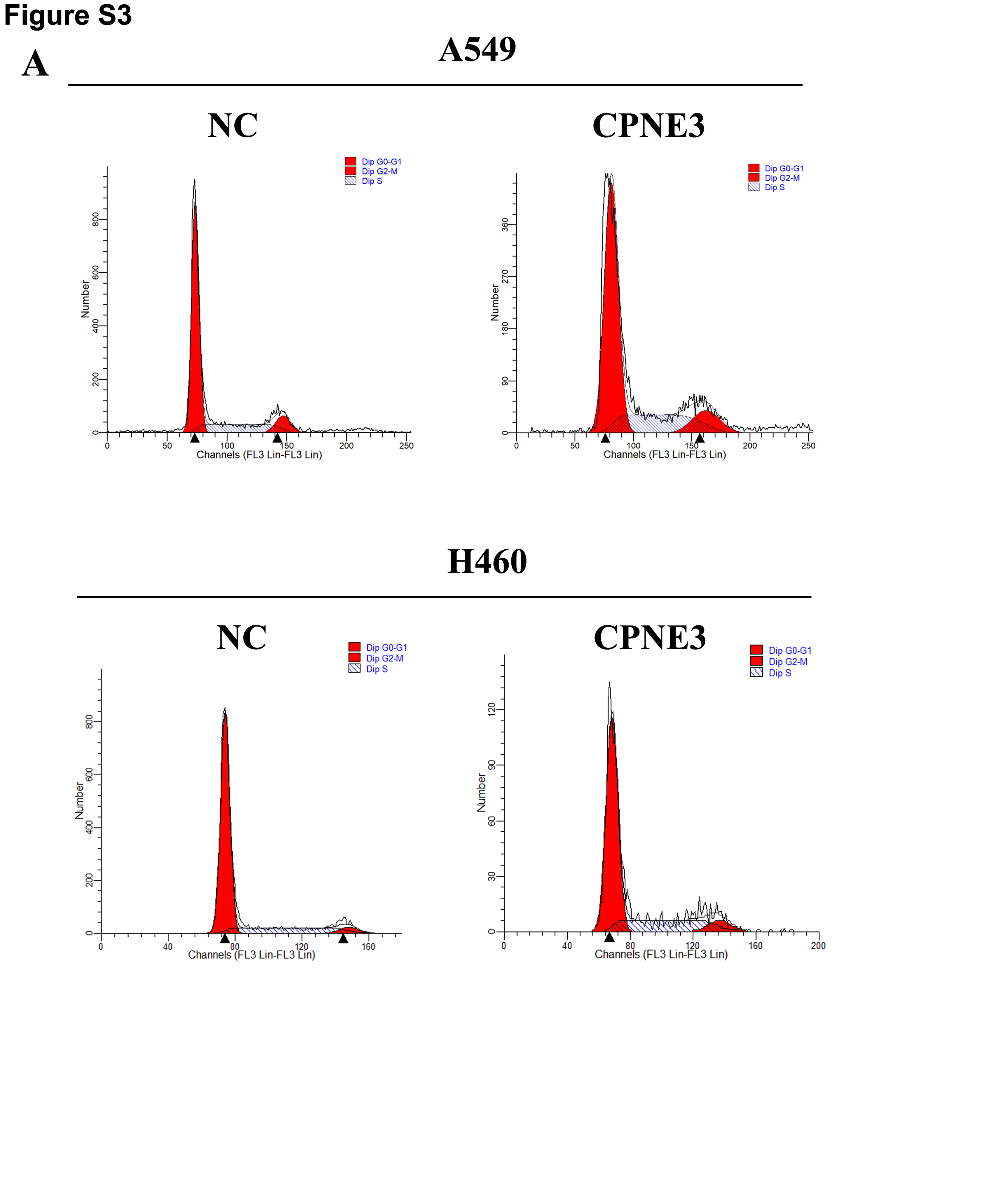

Supplement: Supplementary file 3 — Figure S3: (A) Flow cytometry indicated that after CPNE3‐overexpression, the number of cells in the G0/G1 phase decreased, while the number of cells in the S (DNA synthesis) phase increased significantly. [file JCMM-29-e70926-s006.tif]

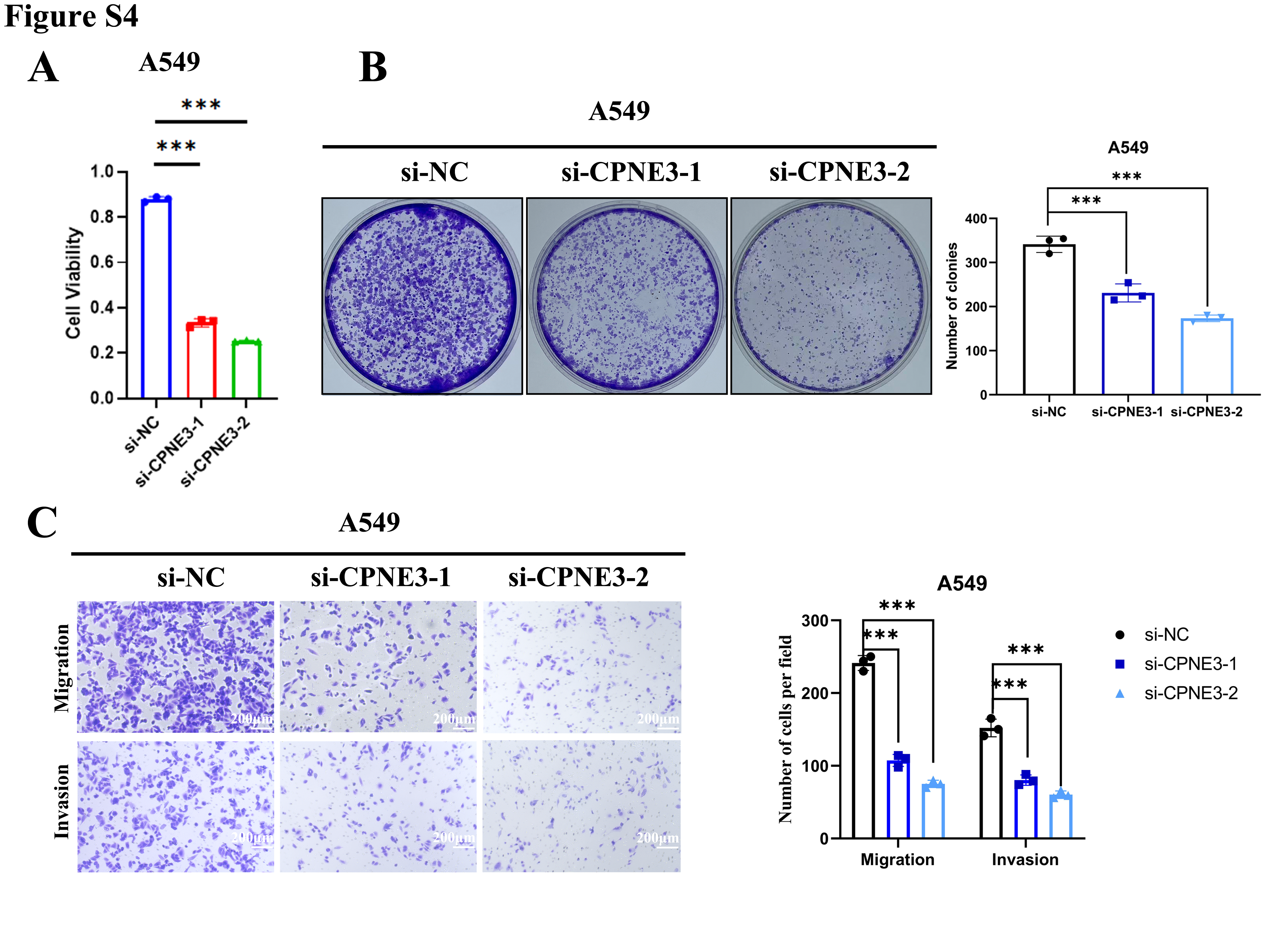

Supplement: Supplementary file 4 — Figure S4: (A) CCK‐8 experiment to detect cell proliferation ability. (B) Clonogenesis assay for detecting cell colony formation ability. (C) Transwell test was used to detect cell migration and invasion ability. *p < 0.05; **p < 0.01; ***p < 0.001. [file JCMM-29-e70926-s009.tif]

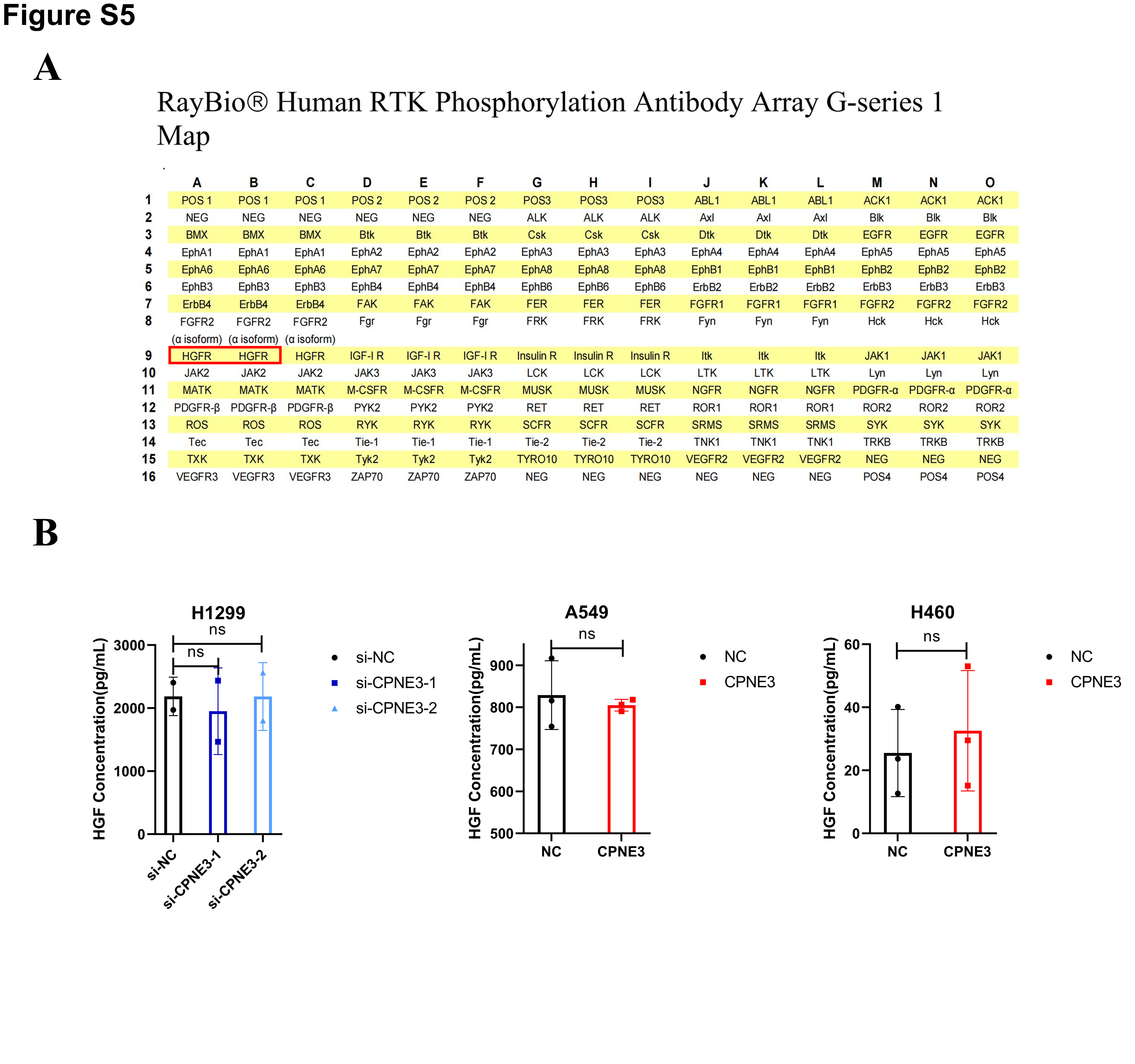

Supplement: Supplementary file 5 — Figure S5: (A) Array map of RayBio Human RTK Phosphorylation Antibody Array G‐series. (B) ELISA assay was performed to detect the effect of CPNE3 expression on HGF concentration in cell supernatant. [file JCMM-29-e70926-s002.tif]

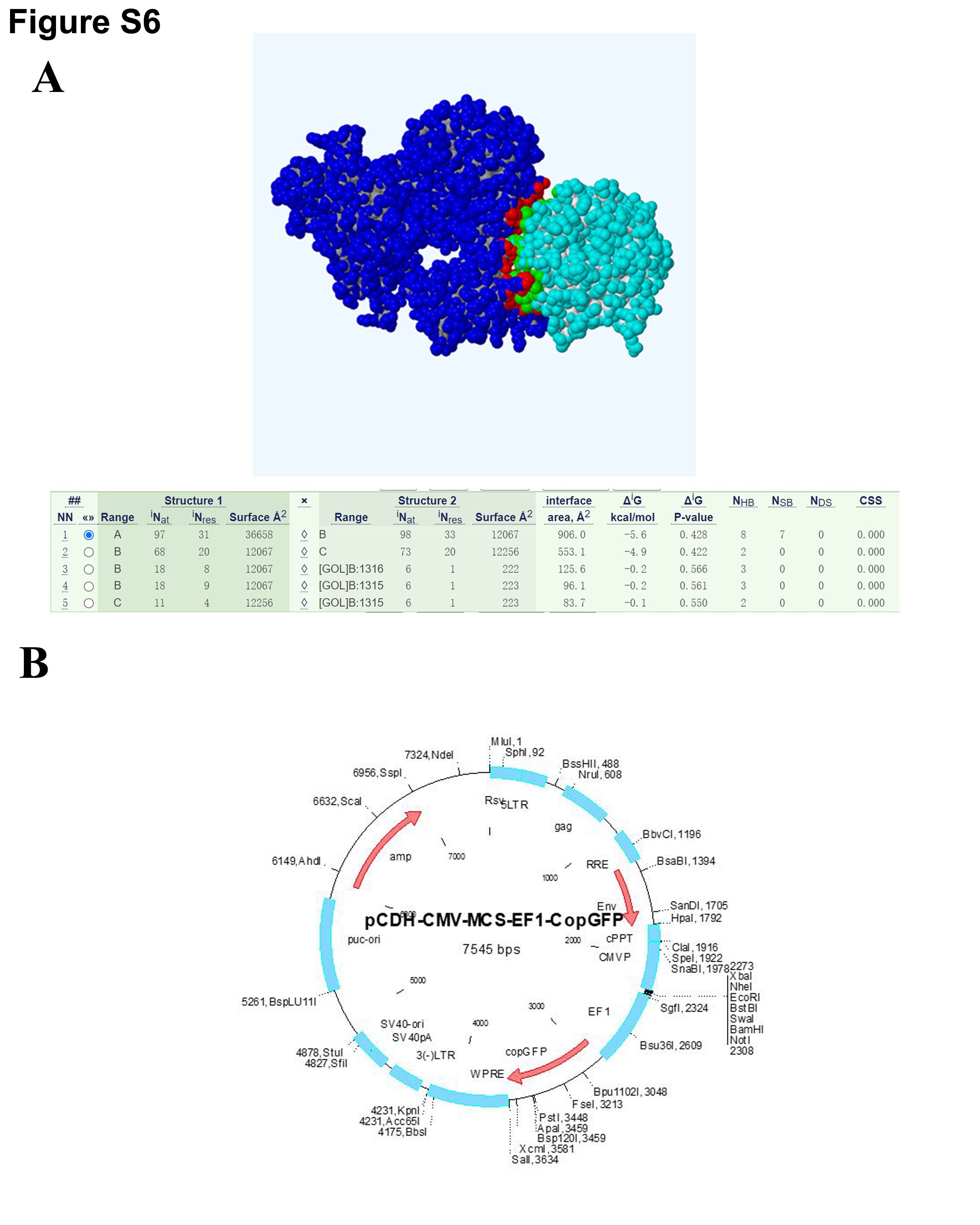

Supplement: Supplementary file 6 — Figure S6: (A) The protein interaction between CPNE3 and RACKl was investigated using the molecular docking dynamic ZDOCK website (https://zdock.umassmed.edu/). (B) Structure of the vector plasmid used in this study. [file JCMM-29-e70926-s001.tif]

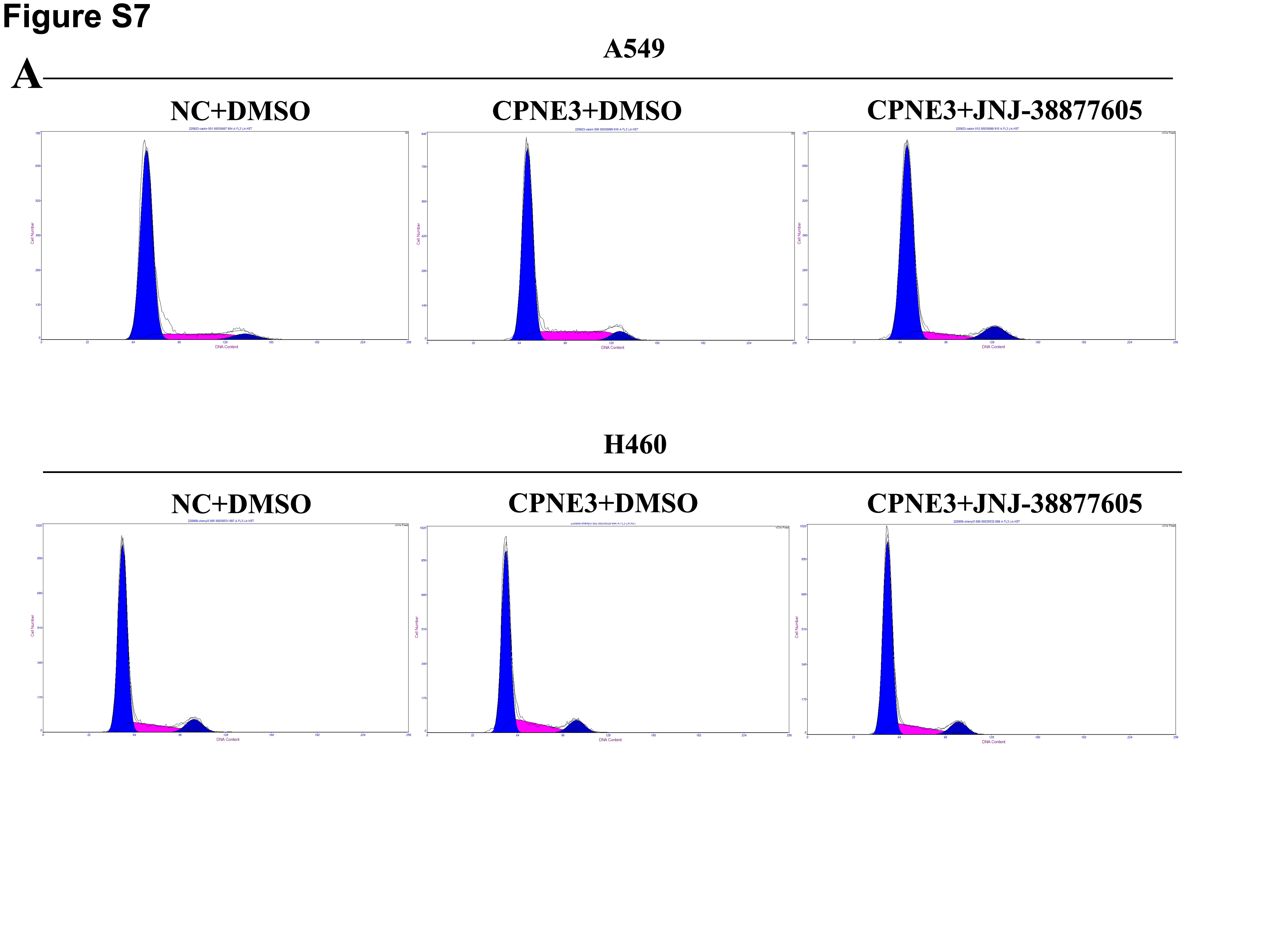

Supplement: Supplementary file 7 — Figure S7: (A) Flow cytometry indicated that JNJ‐38877605 could arrest the cells overexpressing CPNE3 in the G0/G1 phase, while the proportion of cells in the S phase was reduced. [file JCMM-29-e70926-s011.tif]

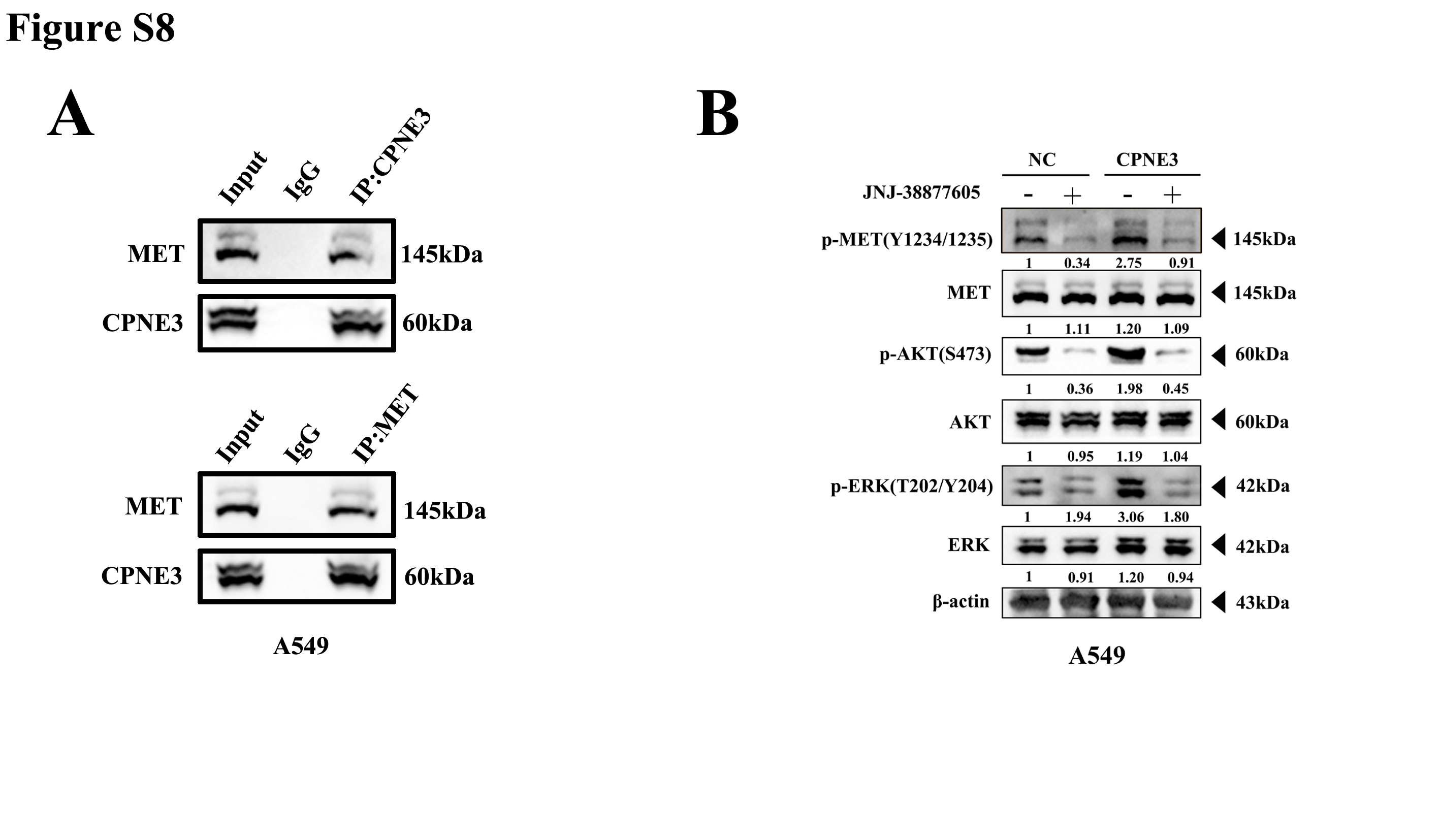

Supplement: Supplementary file 8 — Figure S8: (A) Co‐IP assays demonstrate complex formation between CPNE3 and MET. Lysates from A549 were immunoprecipitated with anti‐CPNE3 or anti‐MET antibodies, followed by immunoblotting with the indicated antibodies. Both forward (IP: CPNE3) and reverse (IP: MET) Co‐IPs confirm specific interaction between CPNE3 and MET, while control IgG shows no precipitation. (B) MET inhibitor JNJ‐38877605 partially reverses CPNE3‐induced AKT and ERK activation. CPNE3‐overexpressing cells were treated with JNJ‐38877605, and phosphorylation levels of AKT and ERK were analysed by Western blot. The results indicate that CPNE3‐driven signalling is dependent on MET activity. [file JCMM-29-e70926-s010.tif]
