# Supplementary material for: Targeting MET Signalling Activated by CPNE3‐RACK1 Interaction Through VWFA Domain to Suppress Lung Cancer Progression
Source: J Cell Mol Med. 2025 Nov 5;29(21):e70926. doi: 10.1111/jcmm.70926 (PMC12587306; doi:10.1111/jcmm.70926)
Supplement: Supplementary file 9 — Table S1: Sequences of siRNAs. [file JCMM-29-e70926-s004.docx]

**Table S1**

Additional file 4: Sequences of siRNAs.

| siRNA | Sense | Anti-sense |
| --- | --- | --- |
| si-NC | 5′-UUCUCCGAACGUGUCACGUTT-3′ | 5′-ACGUGACACGUUCGGAGAATT-3′ |
| si-CPNE3-1 | 5′- GGGACUGGUCAUUCAAGAUTT-3′ | 5′- AUCUUGAAUGACCAGUCCCTT-3′ |
| si-CPNE3-2 | 5′- GCAAUGGAAUCCAAGGCAUTT-3′ | 5′- AUGCCUUGGAUUCCAUUGCTT-3′ |
| si-RACK1 | 5′-CAGAUUGUCUCUGGAUCUCGA-3′ | 5′-UCGAGAUCCAGAGACAAUCUG-3′ |
