# Supplementary material for: Targeting MET Signalling Activated by CPNE3‐RACK1 Interaction Through VWFA Domain to Suppress Lung Cancer Progression
Source: J Cell Mol Med. 2025 Nov 5;29(21):e70926. doi: 10.1111/jcmm.70926 (PMC12587306; doi:10.1111/jcmm.70926)
Supplement: Supplementary file 10 — Table S2: Relationship between clinical characteristics and CPNE3 mRNA expression. [file JCMM-29-e70926-s003.docx]

**Table S2**

|  |  |  |  |  |
| --- | --- | --- | --- | --- |
| **Clinical characteristics** | **n=30** | ***CPNE3* mRNA expression** | | ***P* value** |
|  |  | **low (n=8)** | **high (n=22)** | |
| **Age (years)** |  |  |  |  |
| ≤60 |  | 5 | 7 | 0.13 |
| >60 |  | 3 | 15 |  |
| **Gender** |  |  |  |  |
| Male |  | 1 | 13 | 0.02 |
| Female |  | 7 | 9 |  |
| **Clinical Stage** |  |  |  |  |
| I-II |  | 4 | 8 | 0.50 |
| III |  | 4 | 14 |  |
| **Smoker** |  |  |  |  |
| No |  | 3 | 8 | 0.95 |
| Yes |  | 5 | 14 |  |
