# Supplementary material for: Targeting MET Signalling Activated by CPNE3‐RACK1 Interaction Through VWFA Domain to Suppress Lung Cancer Progression
Source: J Cell Mol Med. 2025 Nov 5;29(21):e70926. doi: 10.1111/jcmm.70926 (PMC12587306; doi:10.1111/jcmm.70926)
Supplement: Supplementary file 11 — Table S3: Relationship between clinical characteristics and CPNE3 protein expression. [file JCMM-29-e70926-s005.docx]

**Tabel S3**

|  |  |  |  |  |
| --- | --- | --- | --- | --- |
| **Clinical characteristics** | **n=45** | **CPNE3 protein expression** | | ***P* value** |
|  |  | **low (n=23)** | **high (n=22)** |  |
| **Age (years)** |  |  |  |  |
| ≤60 |  | 16 | 5 | 0.002 |
| >60 |  | 7 | 17 |  |
| **Gender** |  |  |  |  |
| Male |  | 10 | 10 | 0.894 |
| Female |  | 13 | 12 |  |
| **T** |  |  |  |  |
| T1 |  | 21 | 14 | 0.026 |
| T2-T4 |  | 2 | 8 |  |
| **N** |  |  |  |  |
| N0 |  | 23 | 14 | 0.001 |
| N2 |  | 0 | 8 |  |
| **M** |  |  |  |  |
| M0 |  | 21 | 15 | 0.053 |
| M1 |  | 2 | 7 |  |
